# Calcium activated nucleotidase 1 (CANT1) is critical for glycosaminoglycan biosynthesis in cartilage and endochondral ossification

**DOI:** 10.1016/j.matbio.2018.11.002

**Published:** 2019-08

**Authors:** Chiara Paganini, Luca Monti, Rossella Costantini, Roberta Besio, Silvia Lecci, Marco Biggiogera, Kun Tian, Jean-Marc Schwartz, Céline Huber, Valérie Cormier-Daire, Beth G. Gibson, Katarzyna A. Pirog, Antonella Forlino, Antonio Rossi

**Affiliations:** a Department of Molecular Medicine, Unit of Biochemistry, University of Pavia, Pavia, Italy; b Scuola Universitaria Superiore IUSS, Pavia, Italy; c Department of Biology & Biotechnology, University of Pavia, Pavia, Italy; d Faculty of Biology, Medicine and Health, University of Manchester, Manchester, UK; e Department of Genetics, INSERM UMR1163, Université Paris Descartes-Sorbonne Paris Cité, Institut Imagine, AP-HP, Hôpital Necker Enfants Malades, Paris, France; f Institute of Genetic Medicine, Newcastle University, Newcastle upon Tyne, UK

**Keywords:** BrdU, 5′-bromo-2′-deoxyuridine, CANT1, calcium activated nucleotidase 1, DBQD1, Desbuquois dysplasia type 1, DMMB, dimethylmethylene blue, GAG, glycosaminoglycan, PG, proteoglycan, β-d-xyloside, *p*-nitrophenyl-β-d-xylopyranoside, ΔDi-0S, 3-*O*-β(d-gluc-4-eneuronosyl)-*N*-acetylgalactosamine, ΔDi-4S and ΔDi-6S, derivatives of ΔDi-0S with a sulfate at the 4 or 6 position of hexosamine moiety respectively, Calcium activated nucleotidase 1, Cartilage, Glycosaminoglycan, Growth plate, Proteoglycan, Skeletal dysplasia

## Abstract

Desbuquois dysplasia type 1 (DBQD1) is a chondrodysplasia caused by mutations in *CANT1* gene encoding an ER/Golgi calcium activated nucleotidase 1 that hydrolyses UDP. Here, using *Cant1* knock-in and knock-out mice recapitulating DBQD1 phenotype, we report that CANT1 plays a crucial role in cartilage proteoglycan synthesis and in endochondral ossification. Specifically, the glycosaminoglycan synthesis was decreased in chondrocytes from *Cant1* knock-out mice and their hydrodynamic size was reduced, whilst the sulfation was increased and the overall proteoglycan secretion was delayed. Interestingly, knock-out chondrocytes had dilated ER cisternae suggesting delayed protein secretion and cellular stress; however, no canonical ER stress response was detected using microarray analysis, Xbp1 splicing and protein levels of BiP and ATF4. The observed proteoglycan defects caused deregulated chondrocyte proliferation and maturation in the growth plate resulting in the reduced skeletal growth. In conclusion, the pathogenic mechanism of DBQD1 comprises deregulated chondrocyte performance due to defective intracellular proteoglycan synthesis and altered proteoglycan properties in the extracellular matrix.

## Highlights

• Desbuquois dysplasia type 1 (DBQD1) is a recessive skeletal dysplasia caused by mutations in *CANT1* gene, a Calcium activated nucleotidase of the ER/Golgi.
• The *Cant1* knock-out mouse recapitulates human DBQD1.
• Cant1 is critical for different steps of proteoglycan biosynthesis including glycosaminoglycan chain synthesis, length and sulfation.
• The intracellular GAG synthesis defects cause delayed proteoglycan secretion with ER enlargement.
• In *Cant1* knock-out chondrocytes ER enlargement is not linked to canonical ER stress.
• The proteoglycan defects cause deregulated chondrocyte proliferation and maturation in the growth plate resulting in reduced skeletal growth.

## Introduction

Proteoglycans (PGs) are a complex class of macromolecules ubiquitously distributed in the extracellular matrix (ECM) and on the cell surface. They are composed of a core protein to which a variable number of glycosaminoglycan (GAG) side chains are covalently attached [1]. GAGs are linear polysaccharides classified according to the composition of their disaccharide units in chondroitin sulfate, dermatan sulfate, keratan sulfate and heparan sulfate. In addition, sugar moieties are modified by sulfation at various hydroxyl groups and by epimerization of uronic acid. These features give rise to a tremendous diversity, crucial for the structural properties of the ECM and for a wide range of biological events, including cell signaling, cell proliferation, tissue morphogenesis and growth factor interactions [2,3]. The biosynthesis of GAGs involves a great number of glycosyltransferases, epimerases and sulfotransferases well orchestrated in the Golgi apparatus and, in addition, enzymes and transporters involved in providing the building components such as activated sugars and sulfate. A deficiency or malfunction of any enzyme involved in PG biosynthesis may lead to disorders of varying severity. Most of these disorders affect the skeleton and in some instances the skin [4]. The biosynthesis of defective PGs may affect their charge density or their interactions with other extracellular components, thus affecting the overall ECM structure and properties [5].

According to the most recent nosology and classification of skeletal disorders there are >400 different clinical phenotypes classified in 42 groups [6]; the skeletal abnormalities resulting from defects in GAG synthesis are: chondrodysplasia, short stature, decreased bone density, digit patterning defects, brachydactyly, multiple joint dislocations and advanced carpal ossification.

Desbuquois dysplasia (DBQD) is a rare autosomal recessive chondrodysplasia characterized by short stature, joint laxity, short extremities and round face; the radiological features include “Swedish key” appearance of the proximal femur, advanced carpal and tarsal bone age and progressive scoliosis. Two forms of DBQD have been described: type 1 (DBQD1) with additional hand anomalies (extra ossification centre distal to the second metacarpal, delta phalanx, bifid distal phalanx of the thumb and phalangeal dislocations) and type 2 (DBQD2) without additional hand anomalies [7]. DBQD1 is caused by mutations in *CANT1* gene, encoding for Calcium Activated Nucleotidase 1, while DBQD2 is caused by mutations in *XYLT1* gene, encoding for Xylosyltransferase 1 [8,9]. XYLT1 is involved in the very first step of GAG biosynthesis: it transfers a Xyl residue from UDP-Xyl to the specific serine residues in the newly synthesised core protein of PGs [2]. CANT1 is a nucleotidase present in the endoplasmic reticulum (ER) and Golgi that preferentially hydrolyses UDP to UMP and phosphate [10]. Due to its substrate preference and its localization, it has been suggested that CANT1 might play a role in PG synthesis through the hydrolysis of UDP, a product of glycosyltransferase reactions [8]. UDP removal is essential for the glycosyltransferases, preventing their reaction inhibition and allowing the exchange of UMP with the cytosolic UDP-sugars through an antiporter exchanger. Interestingly, fibroblasts from DBQD1 patients showed reduced PG synthesis, in particular when cells were incubated with β-d-xyloside, a compound that increases GAG synthesis [11].

CANT1 is a member of the apyrase family with sequence homology to apyrases present in the saliva of hematophagous arthropods that hydrolyse extracellular nucleotides, such as ATP and ADP, acting as anti-hemostatic agents [12]. Although the role of apyrases has been elucidated in blood-feeding arthropods, the exact physiological function of CANT1 in human tissues remains to be determined. In 2002, two different groups characterized two forms of the same enzyme, a membrane bound and a soluble secreted form [10,13]. Thus, unlike apyrases found in the blood-feeding arthropods, the substrate preference and subcellular localization of the enzyme suggest very different functions in mammals. In fact, CANT1 is likely involved in the intracellular glycosylation reactions and, in addiction, in protein quality control and folding as demonstrated in neuroblastoma cell lines [14]. Moreover, since uridine nucleotides as well as UDP-sugars are also recognized as extracellular signaling molecules, the presence of the soluble secreted form of CANT1 suggests that the enzyme may also modulate cellular responses to extracellular UDP *via* specific pyrimidinergic receptors (P2Y family) [15].

Overall, results to date suggest that CANT1 has several biological functions *in vitro,* depending on the cell type. Interestingly, mutations in CANT1 leading to a partial or total loss of the enzyme function primarily affect the skeleton *in vivo*, as demonstrated by the clinical phenotype of DBQD1 patients. Unfortunately, since skeletal tissue biopsies from DBQD1 patients are rarely available, a detailed picture of the biochemical, molecular and cellular events in the DBQD1 cartilage and bone, ultimately resulting in dwarfism, progressive joint disease and malformed skeleton, is far from complete.

To provide new insight on the role of CANT1 in the development and homeostasis of the skeleton and to contribute to the understanding of the disease-causing mechanism of *CANT1* mutations in DBQD1, we have generated novel *Cant1* knock-in and knock-out murine strains. We show that both strains present with a skeletal and connective tissue phenotype that recapitulates human DBQD1. Moreover, biochemical studies of knock-out cartilage samples and primary chondrocytes demonstrate that CANT1 defects not only affect GAG synthesis, but also GAG elongation, sulfation and PG secretion.

## Results

### Generation of the *Cant1* knock-in and knock-out mice

**Fig. 1:**
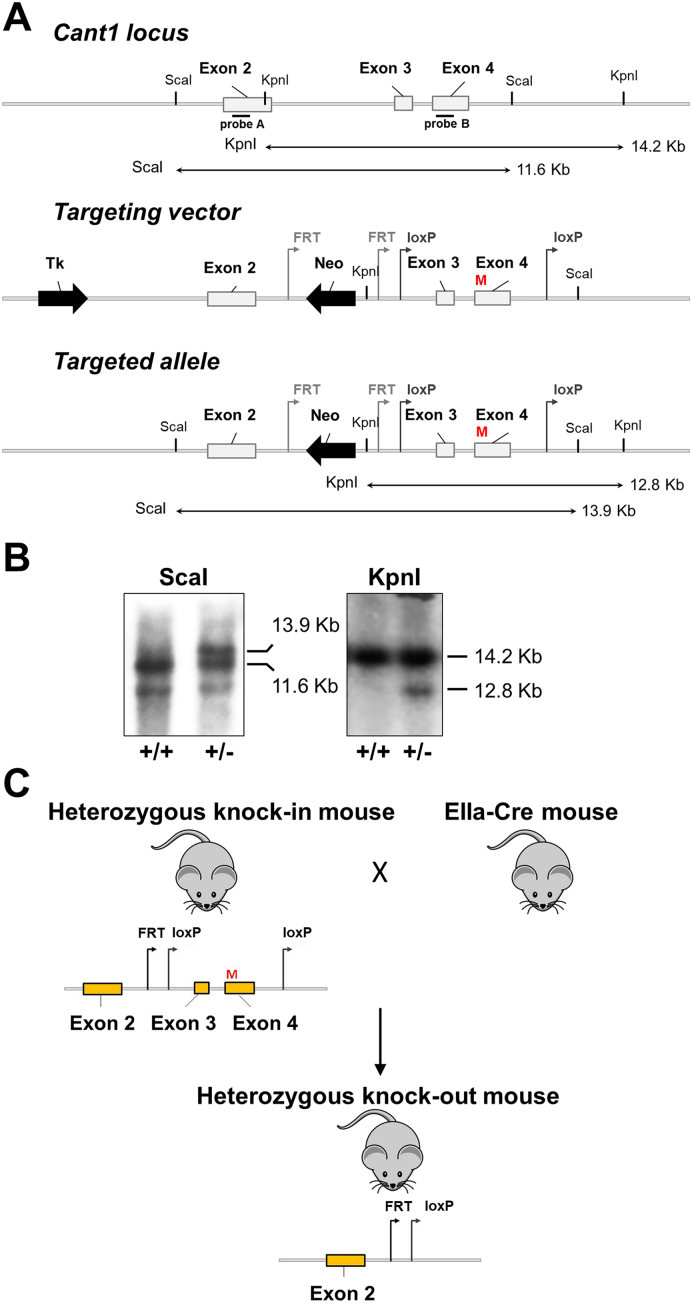
Generation of *Cant1* knock-in and knock-out mice. (A) Schematic representation of *Cant1* locus and targeting vector used for the generation of *Cant1* knock-in (*Cant1*^R302H/R302H^) and knock-out (*Cant1*^−/−^) mice. A c.G905A transition (M) causing the R302H substitution was inserted in *Cant1* exon 4 to generate the *Cant1* knock-in mouse, whilst exon 3 and 4 were flanked by loxP sites to allow the generation of the *Cant1* knock-out mouse by Cre recombinase excision. (B) Southern blot analysis to identify embryonic stem clones that had undergone homologous recombination. The 13.9 kb *ScaI* fragment and the 12.8 kb *KpnI* fragment indicate proper 5′ and 3′ targeting of the *Cant1* locus. (C) Schematic diagram of heterozygous *Cant1* knock-out mouse generation by the excision of exon 3 and 4 obtained mating heterozygous *Cant1* knock-in mice with EIIa-Cre deleter mice (B6.FVB-Tg(EIIa-cre)C5379Lmgd).

We generated transgenic knock-in mice harbouring the p.R302H substitution, which is homologous to the p.R300H substitution in the active site of human CANT1 using a gene targeting approach (Fig. 1). The p.R300H CANT1 mutation has been detected at the homozygous state in two patients of different ethnic origin affected by a moderate form of DBQD1 [8]. To knock-in the missense mutation we introduced a c.G905A transition in a cloned fragment containing exon 4 of the murine *Cant1* gene by site directed mutagenesis. In addition, the gene targeting vector for the generation of the knock-in line was designed to allow a *Cant1* deletion, leading to a knock-out line, by flanking the exon 3 and 4, encoding for the enzyme active site, with loxP sequences (Fig. 1A, C).

The gene targeting vector was electroporated into Sv129/Bl6 embryonic stem (ES) cells. ES clones that had undergone homologous recombination were identified by Southern blot analysis using probes for the 5′ and 3′ end of the targeted region (Fig. 1B). We used two independent ES clones with an euploid karyotype to produce male chimeras; germline transmission of the targeted allele was achieved by breeding the chimeric males with C57Bl/6 J females. Offspring were mated to an Flp deleter mouse strain to remove the positive selection cassette leading to the generation of heterozygous *Cant1* knock-in mice (*Cant1*^+/R302H^ mouse) bearing the R302H missense mutation.

Heterozygous *Cant1* knock-out mice (*Cant1*^+/−^ mouse) were generated by mating heterozygous knock-in animals with a Cre deleter murine strain (Fig. 1C). Deletion of exon 3 and 4 and lack of expression of *Cant1* mRNA in homozygous knock-out mice was confirmed by real time PCR on total RNA from skin of newborn mice (relative quantitation: 1.11 ± 0.16, 0.57 ± 0.12 and 0.0003 ± 0.0001 in wild-type, heterozygous and homozygous mice, respectively; *n* = 3; *P* < 0.001 homozygous knock-out *vs.* heterozygous knock-out and wild-type).

### *Cant1* knock-in and knock-out animals present with skeletal abnormalities reminiscent of human DBQD1 phenotype

*Cant1* knock-in and knock-out mice were morphologically characterized to validate them as animal models of DBQD1. Heterozygous *Cant1* knock-in and knock-out mice did not present with an overt phenotype, similar to human heterozygous carriers of *CANT1* mutations. Specifically, their body weights were normal and no skeletal defects were observed by double staining with alcian blue and alizarin red; for this reason we did not study these animals further.

**Fig. 2:**
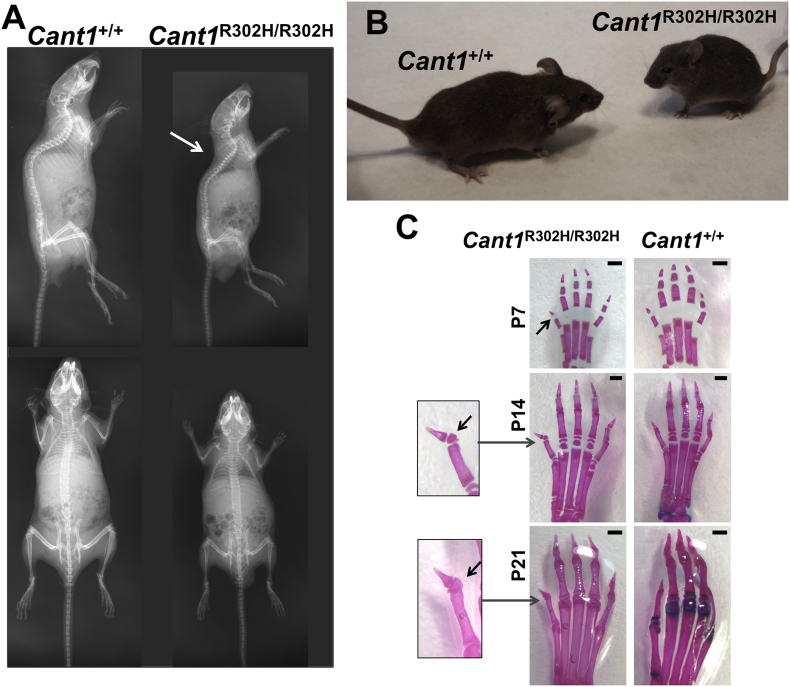
*Cant1* knock-in mice show a skeletal phenotype reminiscent of the human DBQD1. (A) X-ray images of two month old *Cant1* knock-in (*Cant1*^R302H/R302H^) mice showed reduced growth compared with wild type (*Cant1*^+/+^) animals. Moderate thoracic kyphosis (arrow) was present in *Cant1*^R302H/R302H^ mouse. (B) Gross morphology of a two month old *Cant1*^R302H/R302H^ and *Cant1*^+/+^ mice showing growth retardation in the mutant littermate. (C) Alcian blue and alizarin red staining of the hind limb extremities at different ages showed the presence of a delta phalanx (arrow) in *Cant1*^R302H/R302H^ mice. Scale bars = 0.5 mm.

By mating heterozygous knock-in mice, we obtained animals homozygous for the R302H substitution in the CANT1 catalytic domain. At postnatal days (P)60 the homozygous mutant mice demonstrated a skeletal phenotype characterized by reduced size and a moderate thoracic kyphosis (Fig. 2A, B). Moreover, we observed a medial deviation of the first digit in the extremities of *Cant1*^R302H/R302H^ mice with the formation of an additional rudimentary phalanx, so called “delta phalanx” (Fig. 2C), a typical feature in the DBQD1 patients [11].

**Fig. 3:**
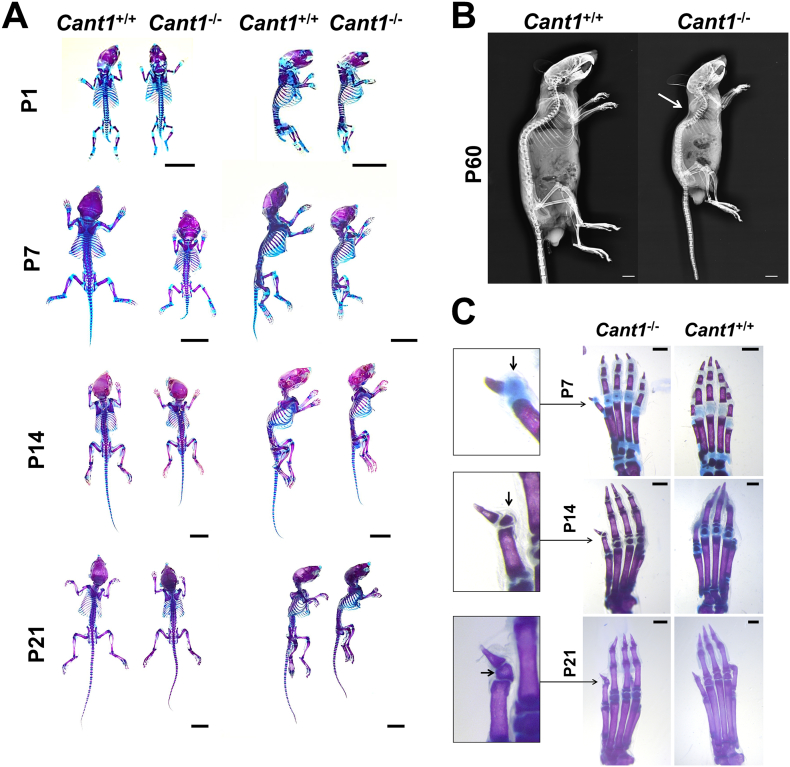
Skeletal phenotype of *Cant1* knock-out mice is reminiscent of the DBQD1 patients' phenotype. (A) Alcian blue and alizarin red staining of full skeletons from birth to P21 showed that *Cant1*^−/−^ mice were smaller compared with age matched wild type (*Cant1*^+/+^) controls. Scale bars = 1 cm. (B) X-ray analysis of two month old (P60) *Cant1*^−/−^ and *Cant1*^+/+^ mice showed reduced growth of *Cant1*^−/−^ compared with wild type animals. Moreover, a moderate thoracic kyphosis (arrow) was observed in *Cant1*^−/−^ mice. Scale bars = 1 cm. (C) Alcian blue and alizarin red staining of the hind limb extremities at different ages revealed the presence of a delta phalanx (arrow) in *Cant1*^−/−^ mice at all ages. Scale bars = 1 mm.

By mating heterozygous knock-out mice, we obtained litters with the normal mendelian ratio of homozygous, heterozygous and wild type animals. We analysed the skeletal phenotype of the knock-out and wild type offspring at different ages from birth to P60 by double staining of cartilage and bone and by X-ray analysis. Alcian blue and alizarin red skeletal preparations from P1 to P21 showed that knock-out (*Cant1*^−/−^) mice were smaller when compared with the wild type littermates (*Cant1*^+/+^), demonstrating a skeletal growth defect (Fig. 3A). In addition, adult *Cant1*^−/−^ mice at P60 were also smaller than the age matched wild type animals (Fig. 3B). Starting from P21, a moderate thoracic kyphosis was evident in the knock-out mice (Suppl. Fig. 1). Moreover, we observed a “delta phalanx” at the extremities of hind limbs of P7, P14 and P21 *Cant1*^−/−^ mice (Fig. 3C), similar to the *Cant1* knock-in mice.

Overall, these data demonstrated a skeletal phenotype with growth retardation and abnormalities in bone extremities reminiscent of the clinical features of DBQD1 in both mouse lines. Since the skeletal phenotype of the knock-in and knock-out mouse lines was similar, in order to study the pathophysiology of *CANT1* mutations in DBQD1 and the role of CANT1 in skeletal homeostasis, we prioritised the deep characterization of the *Cant1*^−/−^ mouse at the biochemical, histological and molecular level.

### Defects in endochondral ossification are present in *Cant1* knock-out mice

**Fig. 4:**
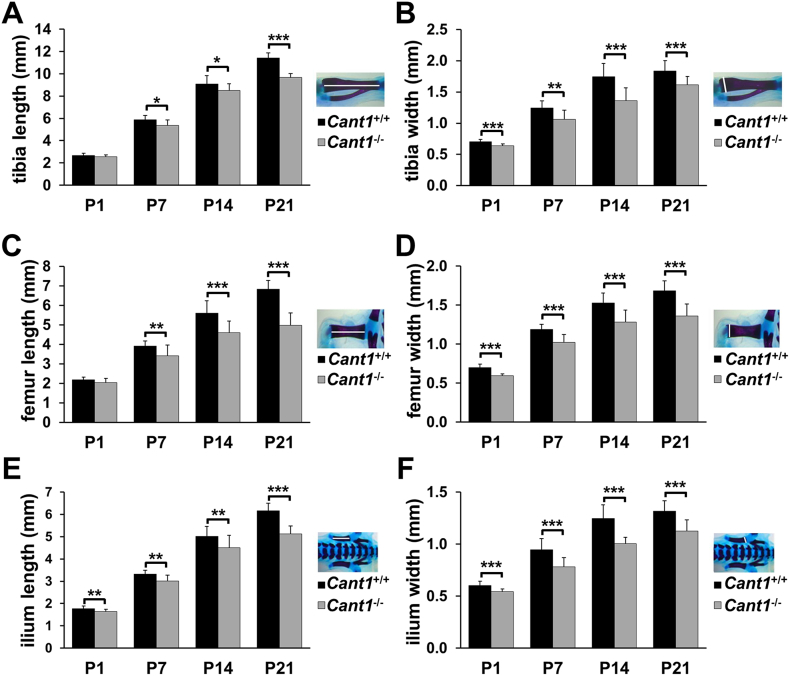
Endochondral ossification is affected in *Cant1*^−/−^ mice. (A – F) Morphometric analysis of alcian blue and alizarin red skeletal preparations. (A) Tibia length was significantly decreased in *Cant1*^−/−^ mice starting from one week of life (P7), whilst (B) tibia width was reduced from birth. Similar changes were observed in (C, D) the femur, whereas (E) both the ilium length and (F) width were significantly decreased in *Cant1*^−/−^ mice from birth. Data are presented as mean ± SD, significance was determined by Student's *t*-test, * *P* < 0.05; ** *P* < 0.01; *** *P* < 0.001; *n* = 5.

We performed morphometric analysis of representative long bones (tibia, femur and ilium) in skeleton preparations of wild type and *Cant1* knock-out mice stained with alcian blue and alizarin red from birth to P21 (Fig. 3A). In knock-out newborn mice the widths of the tibiae and femurs were significantly decreased compared with the age and sex matched wild type bones. At P7, P14 and P21 both length and width of tibiae and femurs were significantly decreased in *Cant1*^−/−^ mice compared with wild type animals. Moreover, a significant reduction in the length and width of the ilium was found in mutant animals compared with wild type littermates at all ages (Fig. 4).

**Fig. 5:**
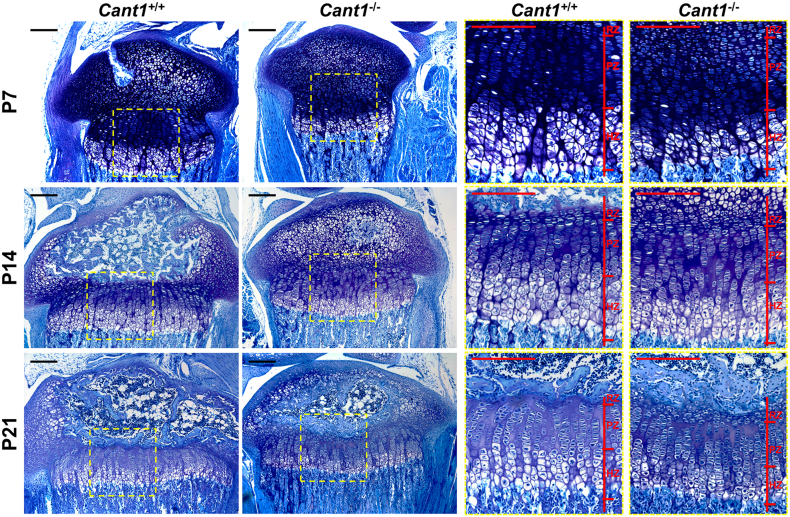
A delay in the formation of secondary ossification centre is observed in *Cant1*^−/−^ mice. Proximal tibia sections of P7, P14 and P21 *Cant1*^−/−^ and *Cant1*^+/+^ mice were stained with toluidine blue. Areas enclosed in the yellow dashed boxes are shown at higher magnification in the right panel. RZ = resting zone; PZ = proliferative zone; HZ = hypertrophic zone; scale bar = 200 μm.

Bone morphometric measurements confirmed the reduced growth of *Cant1*^−/−^ long bones, suggesting a defect in endochondral ossification; we therefore decided to deeply phenotype the tibial growth plate. The overall architecture of the growth plate in *Cant1*^−/−^ mice was maintained, with well-delineated resting, proliferative and hypertrophic zones. In addition, the columnar alignments of chondrocytes in the proliferative and hypertrophic zones were unaffected (Fig. 5). In *Cant1*^−/−^ and wild type animals the height of the growth plate decreased from P7 to P21 following the formation of the secondary ossification centre. However, the height of the proliferative zone in P7 *Cant1*^*−/−*^ growth plates and of the hypertrophic zone in P7 and P14 *Cant1*^−/−^ cartilage was reduced compared with control animals. The area of proliferative and hypertrophic zones showed a similar trend, with a significant difference in both at P7 and in the hypertrophic zone at P14 (Table 1). No disruptions of growth plate organisation were observed in *Cant1*^−/−^ mice at P21.

**Table 1:** Morphometric analysis of the tibia growth plate.

	P7		P14		P21	
	*Cant1*^+/+^	*Cant1*^−/−^	*Cant1*^+/+^	*Cant1*^−/−^	*Cant1*^+/+^	*Cant1*^−/−^
RZ area (μm^2^)	n.d.	n.d.	20,574.93 ± 7008.57	16,979.34 ± 3186.25	26,390.30 ± 9892.46	22,189.23 ± 13,319.88
PZ area (μm^2^)	208,067.47 ± 15,822.06	155,870.54 ± 30,219.71***	222,002.16 ± 71,476.72	186,545.80 ± 40,461.79	154,103.78 ± 27,144.13	135,134.71 ± 35,629.46
HZ area (μm^2^)	156,959.08 ± 13,340.36	89,508.58 ± 12,386.65***	201,043.21 ± 37,338.63	141,589.18 ± 16,126.29**	162,727.19 ± 14,671.09	139,185.84 ± 46,788.93
Relative RZ area (%)	n.d.	n.d.	4.72 ± 1.56	4.93 ± 0.60	7.62 ± 2.66	7.42 ± 2.83
Relative PZ area (%)	56.99 ± 3.07	63.22 ± 5.19*	49.30 ± 8.92	53.63 ± 4.20	44.65 ± 4.49	46.05 ± 4.75
Relative HZ area (%)	43.01 ± 3.07	36.78 ± 5.19*	45.97 ± 7.82	41.44 ± 4.34	47.73 ± 5.70	46.53 ± 4.82
RZ height (μm)			23.87 ± 4.09	23.26 ± 4.53	24.95 ± 5.02	22.10 ± 7.96
PZ height (μm)	268.68 ± 28.06	231.42 ± 30.78*	197.03 ± 63.52	196.06 ± 44.45	113.08 ± 24.53	107.13 ± 25.07
HZ height (μm)	177.06 ± 11.53	128.28 ± 24.22***	168.84 ± 23.58	140.80 ± 10.12*	119.26 ± 11.51	108.23 ± 33.47
Number of column in a 4 × 10^4^ μm^2^ area in the PZ	39.43 ± 6.85	50.89 ± 6.06*	28.08 ± 3.29	32.12 ± 4.07	56.66 ± 6.79	60.05 ± 7.48
Number of cell per column in the PZ	8.57 ± 0.93	6.76 ± 0.46**	9.70 ± 0.54	8.68 ± 0.72	7.15 ± 1.12	7.84 ± 0.71
Height of column in the PZ (μm)	45.56 ± 5.38	33.65 ± 3.20**	62.47 ± 5.10	52.08 ± 6.53*	43.92 ± 4.08	46.33 ± 7.17
Number of column in a 4 × 10^4^ μm^2^ area in the HZ	12.22 ± 2.21	18.30 ± 8.94	12.51 ± 2.38	14.87 ± 3.40	33.97 ± 4.44	38.77 ± 4.03
Number of cell per column in the HZ	10.18 ± 0.93	7.94 ± 0.77**	11.41 ± 1.78	10.29 ± 1.08	7.93 ± 0.92	7.80 ± 1.53
Height of column in the HZ (μm)	136.08 ± 17.64	88.23 ± 7.25***	150.38 ± 8.23	111.14 ± 7.23***	109.11 ± 6.91	95.43 ± 22.67
Height of the most terminal HCs (μm)	25.43 ± 2.77	19.49 ± 0.85**	24.08 ± 0.78	19.42 ± 0.38*	23.27 ± 0.99	19.89 ± 2.05**

All measurements (mean ± SD) were performed on an average of five sections stained with toluidine blue as shown in Fig. 5 and three mice in each group were analysed. Student's *t*-test was performed, * *P* < 0.05; ** *P* < 0.01; *** *P* < 0.001; RZ = resting zone; PZ = proliferative zone; HZ = hypertrophic zone; n.d. = not determined.

The number of columns in a standardized area, the number of cells per column and the column height were measured in proliferative and hypertrophic zones. Interestingly, most differences were observed in the first two weeks of life. At P7, the mutant mice showed a higher number of columns per area in the proliferative zone, but the columns were shorter and with fewer cells compared with wild type growth plates. These changes were also present in the hypertrophic zone at P7, whilst at P14 only the heights of the columns in the proliferative and hypertrophic zones of *Cant1*^−/−^ mice were decreased compared with wild type animals. At P21 no differences between mutant and wild type mice were evident by morphometric analysis (Table 1).

Interestingly, at P7 the relative proliferative zone area was significantly increased in mutant compared with wild type mice and, correspondingly, the relative hypertrophic zone area was markedly decreased, suggesting an imbalance between the two growth plate zones, whilst at other age points no significant differences were observed (Table 1). Longitudinal bone growth is dependent on cell proliferation and on the volume and height that the hypertrophic chondrocytes achieve during terminal differentiation [16,17]. Interestingly, in *Cant1*^−/−^ growth plate the average height of the most terminal hypertrophic chondrocytes was decreased compared with wild type mice at all ages (Table 1). Finally, a delay in the formation of secondary ossification centre was evident in mutant mice at all ages compared with the wild type animals, providing an additional proof of skeletal growth retardation (Fig. 5).

### Proliferation and apoptosis imbalance in growth plates of *Cant1* knock-out mice

To investigate whether the growth plate defects in *Cant1*^−/−^ mice were due to changes in cell proliferation, differentiation and viability, we studied chondrocyte proliferation and apoptosis by 5′-bromo-2′-deoxyuridine (BrdU) labelling and by a TUNEL assay, respectively.

**Fig. 6:**
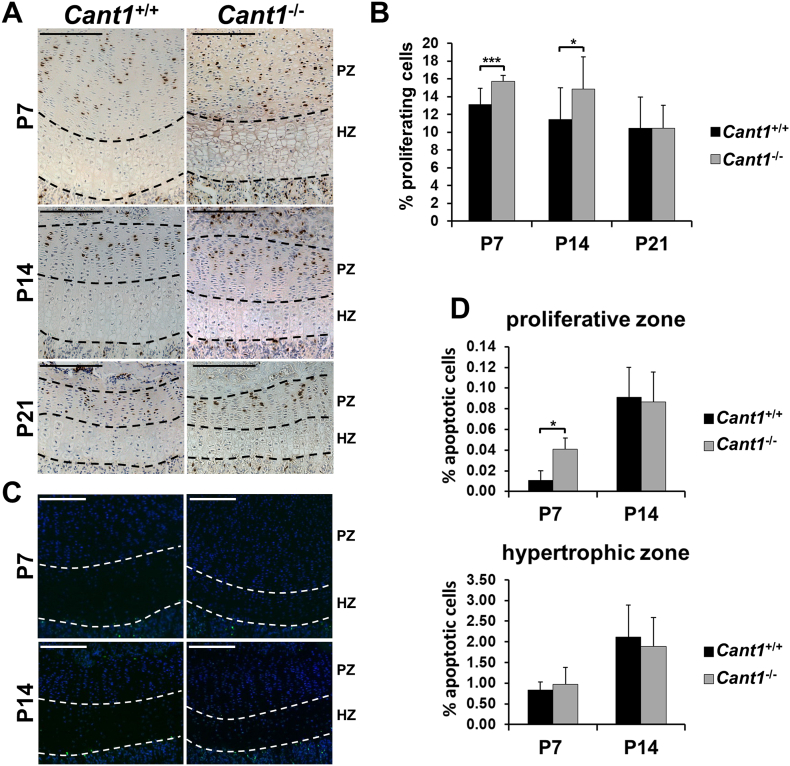
Proliferation and apoptosis are imbalanced in *Cant1*^−/−^ growth plate. (A) BrdU assay on proximal tibia sections of *Cant1*^−/−^ and *Cant1*^+/+^ mice. BrdU positive cells (brown) were only found in the proliferative zone. PZ = proliferative zone; HZ = hypertrophic zone; scale bar = 200 μm. (B) The percentage of proliferating chondrocytes was higher in P7 and P14 *Cant1*^−/−^ proliferative zone. An average of five sections per animal were analysed. Data are reported as mean ± SD and significance was determined by Student's *t*-test, * *P* < 0.05; *** *P* < 0.001; *n* = 3. (C) Fluorescent TUNEL assay on proximal tibia sections of *Cant1*^−/−^ and *Cant1*^+/+^ mice. TUNEL positive cells showed a green nucleus profile, while total cells were stained in blue by DAPI. PZ = proliferative zone; HZ = hypertrophic zone; scale bar = 200 μm. (D) The percentage of apoptotic chondrocytes was increased only in P7 *Cant1*^−/−^ proliferative zone. An average of five sections per animal was analysed. Data are reported as mean ± SD and Student's t-test was performed, * *P* < 0.05; *n* = 3.

BrdU-labelled cells were detected only in the proliferative zone and the ratio of proliferating cells (BrdU positive cells) to the total cell number (haematoxylin positive cells) was calculated. In *Cant1*^−/−^ mice the percentage of proliferating chondrocytes was significantly increased compared with wild type animals at P7 (15.72 ± 0.66% and 13.12 ± 1.84%, respectively; *P* < 0.001, *n* = 3) and P14 (14.87 ± 3.60 and 11.43 ± 3.55%, respectively; *P* < 0.05, n = 3), whilst no differences were detected in P21 *Cant1*^−/−^ mice (Fig. 6A, B).

We measured apoptosis in the proliferative and hypertrophic zones of *Cant1*^−/−^ and wild type mice at P7 and P14. The percentage of apoptotic chondrocytes in the proliferative zone of P7 *Cant1*^−/−^ mice was increased compared with wild type mice (0.04 ± 0.01% and 0.01 ± 0.01%, respectively; *P* < 0.05, n = 3), whereas no difference was detected in the hypertrophic zone of P7 and P14 mice (Fig. 6C, D).

Overall, these results demonstrated an imbalance between proliferation and apoptosis in *Cant1*^−/−^ growth plate consistent with the defects detected by morphometric analysis in the proliferative and hypertrophic zones (Table 1).

### Proteoglycan synthesis is reduced in *Cant1* knock-out mice

It has been hypothesised that CANT1 plays a role in PG synthesis through the hydrolysis of UDP produced by glycosyltransferases. We therefore decided to study the PG metabolism and synthesis in primary chondrocyte cultures from rib cartilage of mutant and wild type mice at P4 by metabolic labelling with ^35^S-sulfate.

**Fig. 7:**
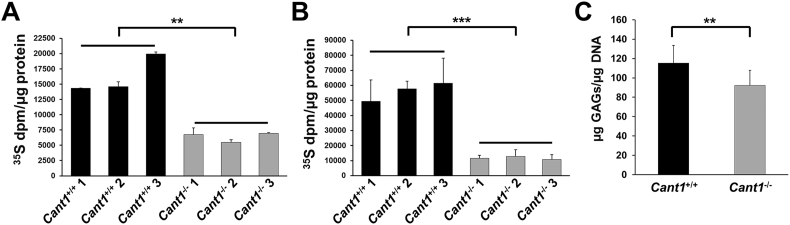
Proteoglycan synthesis is reduced in *Cant1*^−/−^ mice. (A) Rib chondrocyte cultures were metabolically labelled with ^35^S-sulfate for 24 h in basal medium. The amount of newly synthesised PGs was determined on the basis of ^35^S-activity and normalised to the protein content, demonstrating that *Cant1*^−/−^ chondrocytes produced less PGs compared with wild type cells. Data are representative of two independent experiments and reported as mean ± SD. Significance was determined by Student's *t*-test; ** *P* < 0.01. (B) Rib chondrocyte cultures were metabolically labelled with ^35^S-sulfate for 24 h in basal medium containing 1 mM *p*-nitrophenyl-β-d-xylopyranoside. Samples were analysed as described in 7A, demonstrating that the reduced PG synthesis in *Cant1*^−/−^ chondrocytes was more evident in presence of β-d-xyloside. Data are representative of two independent experiments and reported as mean ± SD. Student's t-test was performed, *** *P* < 0.001. (C) The GAG content in femoral head cartilage from 4 day old mice was analysed by dimethylmethylene blue assay and normalised to DNA content, showing reduced GAG content in *Cant1*^−/−^ cartilage. Data are representative of three independent experiments and reported as mean ± SD. Student's t-test was performed, ** *P* < 0.01; *n* = 10.

In basal conditions (MEM), PG synthesis was reduced in *Cant1*^−/−^ chondrocytes compared with wild type cells (*P* < 0.01, n = 3). This defect was even more enhanced when the cells were metabolically labelled in MEM containing *p*-nitrophenyl-β-d-xylopyranoside (β-d-xyloside) (*P* < 0.001, n = 3) (Fig. 7A, B).

To evaluate if CANT1 defect affects PG synthesis *in vivo*, we analysed the GAG content of cartilage from mutant and wild type mice. Femoral head cartilage of P4 mice was digested with proteinase K to remove proteins and release GAGs. The GAG content, quantified by the dimethylmethylene blue (DMMB) assay and normalised to DNA, was reduced in *Cant1*^−/−^ mice compared with control animals (*P* < 0.01, *n* = 10) (Fig. 7C).

In conclusion, the PG content was reduced in *Cant1*^−/−^ chondrocyte cultures and in *Cant1*^*−/−*^ cartilage.

### Glycosaminoglycans are oversulfated and show reduced hydrodynamic size in *Cant1* knock-out cartilage

**Fig. 8:**
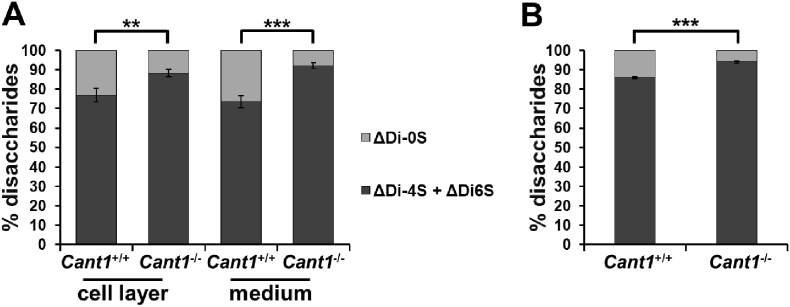
Chondroitin sulfate proteoglycans are oversulfated in *Cant1*^−/−^ mice. (A) Sulfation of chondroitin sulfate proteoglycans extracted from cell layer and medium of *Cant1*^−/−^ and *Cant1*^+/+^ chondrocyte cultures was analysed by reverse-phase HPLC after digestion with chondroitinase ABC and ACII. The percentage of monosulfated disaccharides (ΔDi-4S and ΔDi-6S) was significantly increased in *Cant1*^−/−^ cells compared with wild type controls. Data are reported as mean ± SD and significance was determined by Student's *t*-test, **P < 0.01, ***P < 0.001; *n* = 3. (B) Chondroitin sulfate proteoglycans were extracted from P4 *Cant1*^−/−^ and *Cant1*^+/+^ femoral head cartilage and their sulfation was analysed by reverse-phase HPLC after digestion with chondroitinase ABC and ACII. The percentage of monosulfated disaccharides (ΔDi-4S and ΔDi-6S) was significantly increased and correspondingly the percentage of non-sulfated disaccharides (ΔDi-0S) was reduced in *Cant1*^−/−^ cartilage compared with wild types. Data are reported as mean ± SD and Student's *t*-test was performed, ***P < 0.001; *n* = 6.

To assess whether CANT1 defect affects other structural properties of PGs, we analysed GAG sulfation in primary chondrocytes by HPLC analysis of chondroitin sulfate disaccharides. The percentage of monosulfated disaccharides (ΔDi-4S and ΔDi-6S) relative to the total amount of disaccharides (ΔDi-0S, ΔDi-4S and ΔDi-6S) was increased in *Cant1*^−/−^ chondrocytes compared with *Cant1*^+/+^ cells, in both cell layer (88.43 ± 1.91% and 76.92 ± 3.47%, respectively; *P* < 0.01, *n* = 3) and medium fractions (92.22 ± 1.36% and 73.62 ± 3.03%, respectively; *P* < 0.001, n = 3), demonstrating chondroitin sulfate oversulfation in *Cant1*^−/−^ chondrocytes compared with wild type cells (Fig. 8A).

To confirm this result *in vivo*, we analysed sulfation of chondroitin sulfate from femoral head cartilage of P4 *Cant1*^−/−^ and wild type mice by HPLC. There was a significant increase in the relative amount of monosulfated disaccharides in *Cant1*^−/−^ mice compared with wild type controls (94.05 ± 0.56% and 86.01 ± 0.49%, respectively; *P* < 0.001, *n* = 6), indicating PG oversulfation in *Cant1*^−/−^ mouse cartilage (Fig. 8B).

To analyse whether the increased sulfation in mutant mice was due to an overexpression of chondroitin sulfotransferases, we performed expression studies using RNA from rib cartilage of P4 *Cant1*^−/−^ and wild type mice on an Affymetrix genechip microarray. No significant differences between *Cant1*^−/−^ and wild type animals were detected with a fold expression change set to ±2 (Suppl. Table 1).

**Fig. 9:**
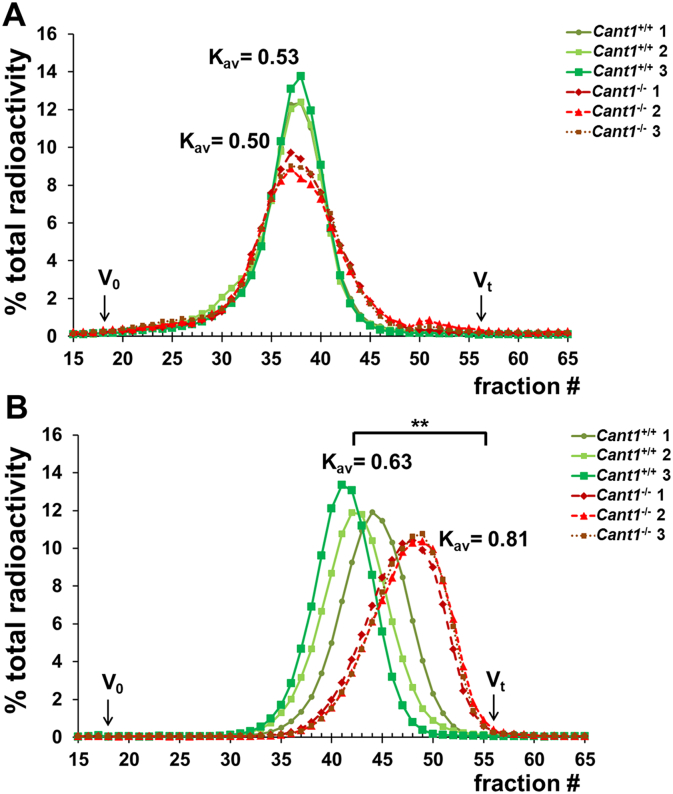
GAG hydrodynamic size is reduced in *Cant1*^−/−^ mice. (A) Chondrocyte cultures were metabolically labelled with ^35^S-sulfate for 24 h in basal medium. GAGs were released from PGs by β-elimination and their hydrodynamic size was analysed by gel filtration chromatography. GAG hydrodynamic size of *Cant1*^−/−^ chondrocytes was comparable with *Cant1*^+/+^ cells even if a slight peak broadening toward the total volume (V_t_) was observed. Data are representative of two independent experiments. (B) *Cant1*^−/−^ and *Cant1*^+/+^ chondrocyte cultures were metabolically labelled with ^35^S-sulfate in basal medium containing 1 mM *p*-nitrophenyl-β-d-xylopyranoside, an enhancer of GAG synthesis. GAG hydrodynamic size was analysed by gel filtration chromatography. A marked shift toward the V_t_ was found in *Cant1*^−/−^ chromatograms demonstrating that *Cant1*^−/−^ GAGs have reduced size compared with wild type controls. Data are representative of two independent experiments and K_av_ is reported as mean. Significance was determined by Student's t-test, ** *P* < 0.01.

Since both the GAG content and the sulfation were altered in cartilage and in chondrocyte cultures from *Cant1*^−/−^ mice, we also considered the hydrodynamic size of the GAGs. When chondrocytes were incubated in basal medium (MEM), the hydrodynamic size of GAG chains in *Cant1*^−/−^ and *Cant1*^+/+^ cells was similar (K_av_ 0.50 and 0.53, respectively; *n* = 3), even if a slight peak broadening toward the total volume (V_t_) was evident in *Cant1*^−/−^ chromatograms, indicating a fraction of GAGs with lower molecular mass compared with wild type controls (Fig. 9A). Interestingly, when cells were metabolically labelled in MEM containing β-d-xyloside, GAGs from *Cant1*^−/−^ chondrocytes showed a significant shift toward the V_t_ compared with wild type cells (K_av_ 0.81 ± 0.02 and 0.63 ± 0.05, respectively; *P* < 0.01, n = 3), (Fig. 9B). This result demonstrated the smaller size of newly synthesised GAGs in *Cant1*^−/−^ chondrocytes compared with control cells when oligosaccharide synthesis was enhanced.

In summary, these results demonstrated that when CANT1 was impaired, beyond reduced synthesis of PGs, GAG sulfation pattern and hydrodynamic size were also altered.

### Proteoglycan secretion is reduced in *Cant1* knock-out mice

Results described above demonstrated defects in different steps of PG biosynthesis in *Cant1*^−/−^ chondrocytes that could impact on PG secretion. We therefore investigated PG secretion by a pulse chase experiment in primary rib chondrocytes.

**Fig. 10:**
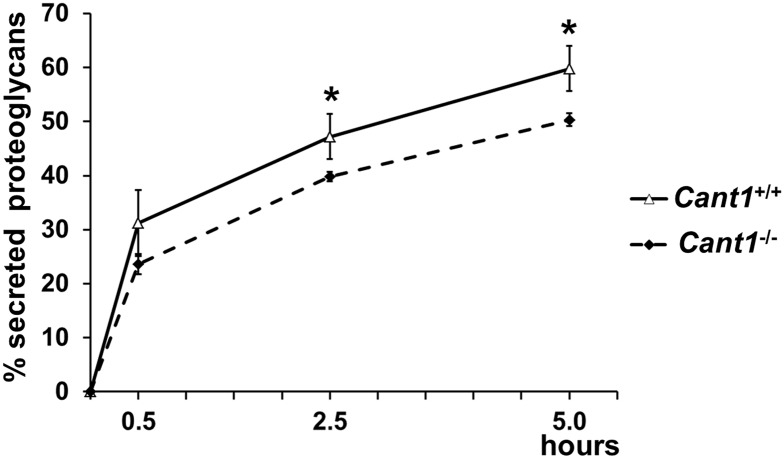
Proteoglycan secretion is delayed in *Cant1*^−/−^ mice. Chondrocyte cultures were metabolically labelled with ^35^S-sulfate for 2 h and cells were harvested after 0.5, 2.5 and 5 h. The percentage of ^35^S-PGs in the medium to total labelled PGs (in medium and cell layer) was calculated and showed a delayed secretion of PGs in *Cant1*^−/−^ chondrocytes. Data are reported as mean ± SD and significance was determined by Student's *t*-test, * *P* < 0.05; *n* = 3.

A significant impairment of PG secretion was demonstrated in mutant chondrocytes compared with wild type cells (*P* < 0.05, *n* = 3) (Fig. 10).

**Fig. 11:**
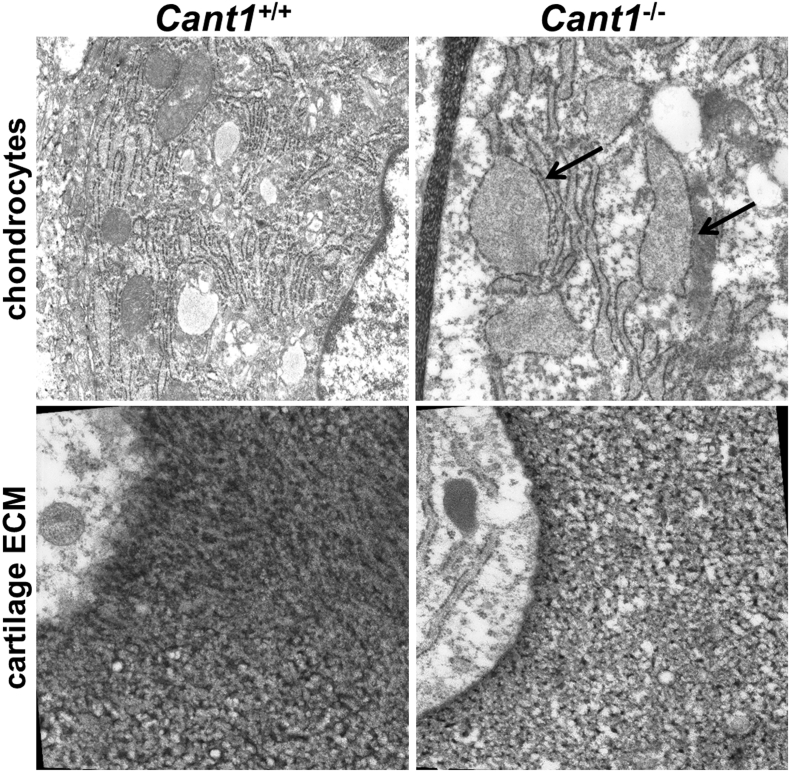
ER enlargement is present in *Cant1*^−/−^ cartilage. Femoral head cartilage sections of P4 *Cant1*^−/−^ and *Cant1*^+/+^ mice were analysed by TEM. In *Cant1*^−/−^ samples the presence of ER enlargement in chondrocytes (arrows) and a less dense cartilage ECM compared with wild type sections were observed. Three animals for each genotype were analysed. Magnification in chondrocyte images: 12,000× and in cartilage images: 20,000×.

The defects in PG synthesis and secretion, detected *in vitro*, suggested the study of chondrocytes at the ultrastructural level by transmission electron microscopy (TEM). In *Cant1*^−/−^ cartilage sections of P4 mice, chondrocytes showed ER enlargement with retained proteinaceous material, as previously shown in patient fibroblasts [8] (Fig. 11). Interestingly, the *Cant1*^−/−^ femoral head cartilage sections at P4 also showed a less dense ECM compared with wild type samples (Fig. 11), consistent with reduced GAG content in *Cant1*^−/−^ cartilage observed by the DMMB assay (Fig. 7C).

Taken together these results further confirmed the biochemical data demonstrating defects in PG metabolism and secretion in *Cant1*^*−/−*^ cartilage.

### Despite the dilated ER cisternae, ER stress is not present in *Cant1* knock-out chondrocytes

The enlargement of the ER observed by TEM analysis in mutant mice suggested protein retention and possibly ER stress in *Cant1*^−/−^ chondrocytes. Indeed, retention of proteins and accumulation of unfolded proteins in the ER leading to increased ER volume and activation of the unfolded protein response (UPR) have been previously demonstrated in several skeletal disorders [18,19]. Therefore, we performed expression studies in rib cartilage of P4 *Cant1*^−/−^ and wild type mice using an Affymetrix genechip microarray in order to investigate ER stress and UPR activation in *Cant1*^−/−^ chondrocytes. The analysis of microarray data, focused on genes related to ER stress and UPR, indicated no differences between *Cant1*^−/−^ and wild type animals (Suppl. Table 2). These results suggested that the UPR was not activated in *Cant1*^−/−^ chondrocytes.

**Fig. 12:**
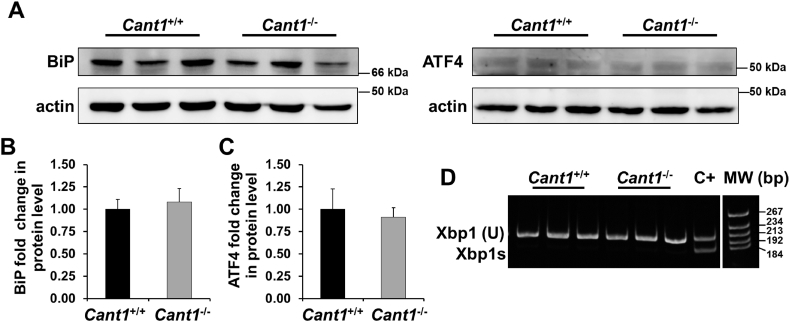
Canonical ER stress is not present in *Cant1*^−/−^ chondrocytes. (A) BiP and ATF4 western blot of *Cant1*^−/−^ and *Cant1*^+/+^ chondrocytes. Actin was used as internal control and data are representative of three independent experiments. (B) BiP protein level of *Cant1*^−/−^ and *Cant1*^+/+^ chondrocytes. The intensity of the wild type band was set to one and the expression of mutant samples was expressed as fold change. Data are representative of three experiments and reported as mean ± SD. Significance was determined by Student's *t*-test, *P* > 0.05; *n* = 3. (C) ATF4 protein level of *Cant1*^−/−^ and *Cant1*^+/+^ chondrocytes. The intensity of the wild type band was set to one and the expression of mutant samples was expressed as fold change. Data are representative of three experiments and reported as mean ± SD. Student's t-test was performed, *P* > 0.05; n = 3. (D) The spliced form of Xbp1 (Xbp1s) was analysed by RT-PCR and results were separated on acrylamide gel. Only the unspliced form of Xbp1 (U) was present in both *Cant1*^−/−^ and *Cant1*^+/+^ chondrocytes demonstrating that ER stress was not present. Positive control (C+): wild type chondrocytes incubated with tunicamycin overnight to induce ER stress showed both unspliced and spliced form of Xbp1.

To confirm these data at the protein level, we analysed the expression of Binding immunoglobulin Protein (BiP), a master regulator of the UPR, and of Activating Transcription Factor 4 (ATF4), a crucial UPR transcription factor, by western blot of cell lysates from *Cant1*^−/−^ and wild type primary rib chondrocyte cultures. Western blots showed no difference in the levels of BiP and ATF4 between *Cant1*^−/−^ and wild type cells (Fig. 12A, B, C), supporting the expression studies.

The activation of the Inositol-Requiring Enzyme 1 (IRE1) branch of UPR results in splicing of X-box binding protein 1 (Xbp1) mRNA to the Xbp1s form that is translated into a transcription factor [20]. In both *Cant1*^−/−^ and wild type chondrocytes only the unspliced form of Xbp1 was found (Fig. 12D), providing a further proof that UPR was not activated in *Cant1*^−/−^ chondrocytes.

Overall, these results demonstrated that ER stress was not present in *Cant1*^−/−^ mice, even though ER enlargement and delayed PG secretion were observed (Fig. 10, Fig. 11).

## Discussion

The molecular mechanisms underlying the genetic diseases are often quite complex and involve deregulation and misfunction of several genes. Even for monogenic disorders understanding the genotype to phenotype correlation is difficult when the gene involved is poorly understood. A *Cant1*^*−/−*^ mouse was generated by the excision of exon 3 and 4 encoding for the active site of the enzyme in order to define the physiological role of CANT1 and to better understand the pathophysiology of DBQD1 (Fig. 1). The chondrodysplastic phenotype in mutant mice recapitulated that of the DBQD1 patients. The skeletal growth of mutant mice was reduced compared with wild types and a “delta phalanx”, a typical hand abnormality described in DBQD1 patients [8], was present at the extremities of *Cant1*^*−/−*^ mice (Fig. 3).

### Endochondral ossification defects

Morphometric analysis of long bones demonstrated reduced size and growth retardation in mutant mice (Fig. 4). In *Cant1*^*−/−*^ mice, the height and area of the proliferative and hypertrophic zones of the growth plate were markedly reduced (Table 1). Chondrocyte proliferation was increased in *Cant1*^*−/−*^ mice compared with the wild type animals at P7 and P14, whilst a higher percentage of apoptotic chondrocytes was only detected in the proliferative zone of P7 *Cant1*^*−/−*^ mice (Fig. 6). Thus, the high chondrocyte proliferation rate in *Cant1*^*−/−*^ mice was balanced by the increased apoptosis in the first week of life when significant differences in growth plate morphology were observed.

During hypertrophy, chondrocytes undergo terminal differentiation and their height and volume increase fourfold and tenfold, respectively [16,17]. In *Cant1*^*−/−*^ mice we observed an impairment of the terminal chondrocyte differentiation at P7, P14 and P21, indicated by the decreased height of the most terminal hypertrophic chondrocytes compared with wild type animals (Table 1).

The growth plate defects described in *Cant1*^−/−^ mice are consistent with the putative role of CANT1 in GAG biosynthesis. A disorganized growth plate as well as alterations in the orientation of chondrocyte columns and in chondrocyte function has previously been described in animal models with altered synthesis, length or sulfation of GAGs [[21], [22], [23], [24]]. Thus, we speculate that the structural defects of PGs present in *Cant1*^−/−^ ECM can lead to altered binding of growth factors, thus increasing the proliferation of growth plate chondrocytes and impairing chondrocyte differentiation. Alternatively, the high cell proliferation rate could be a compensative process in order to restore defects caused by impaired terminal differentiation of chondrocytes.

### Glycosaminoglycan synthesis defects

Since PGs play a crucial role in the cartilage growth plate development and homeostasis [25], we therefore investigated the role of CANT1 in PG biosynthesis using mouse tissues and primary cultures of rib chondrocytes from *Cant1* mutant and wild type mice. *Cant1*^*−/−*^ chondrocytes synthesised less PGs compared with the wild type cells both *in vitro* and *in vivo* (Fig. 7); moreover, GAG chains in the *Cant1*^*−/−*^ chondrocytes were smaller in size and oversulfated compared with wild type controls (Fig. 8A, 9). Increased GAG sulfation was further confirmed in the cartilage of mutant mice (Fig. 8B). The defects in different intracellular steps of GAG biosynthesis caused a reduction of PG secretion in *Cant1*^*−/−*^ chondrocytes compared with wild type cells (Fig. 10). Interestingly, we demonstrated that in the absence of CANT1, GAG chains are oversulfated both *in vitro* and *in vivo,* even though increased expression of sulfotransferases was excluded by microarray analysis (Suppl. Table 1). Thus, we postulate that the oversulfation of chondroitin sulfate chains in cartilage of mutant mice is caused by the delay of PG secretion. In particular, we hypothesise that PGs spend more time in the Golgi apparatus of the *Cant1*^*−/−*^ chondrocytes, where sulfotransferases have more time to catalyse GAG sulfation. This observation parallels what has been observed in the dominant forms of Osteogenesis Imperfecta, in which several glycine substitutions in type I collagen triple helix cause a delay in triple helix folding, allowing a longer activity of hydroxylases and glycosyltransferases of the ER and leading to increased level of post-translational modifications [26,27].

Reduced PG synthesis detected in mutant mice was previously observed in fibroblast cultures from DBQD1 patients [11], thus confirming the phenotypic similarities of *Cant1*^*−/−*^ mice with the disorder in humans at the biochemical level. Overall, these data provide a definitive evidence of CANT1 involvement in PG biosynthesis in cartilage.

### ER enlargement and potential cellular stress

To further investigate the molecular basis of the defect in PG secretion, sections of mutant cartilage were analysed by TEM. *Cant1*^*−/−*^ chondrocytes showed dilated ER cisternae with retained electrodense material, suggesting intracellular protein retention (Fig. 11). This finding further validated *Cant1*^*−/−*^ mice as a model of DBQD1, since ER enlargement has previously been reported in patients' fibroblasts [8]. Delayed PG secretion and ER enlargement suggested that ER stress might contribute to the skeletal phenotype. ER stress often triggers a cell response, the UPR, involving the expression of specific transcription factors and chaperones [28]. However, the expression of the main ER stress markers was normal in *Cant1*^*−/−*^ mice (Suppl. Table 2). This finding was supported by normal protein levels of BiP, a master regulator of the UPR [28], and of ATF4 (downstream of Protein kinase R-like ER Kinase, PERK); in addition the alternatively spliced form of Xbp1 mRNA (downstream of IRE1) was not detected in *Cant1*^*−/−*^ cartilage (Fig. 12). Overall, these data support ER enlargement without canonical ER stress in chondrocytes, when CANT1, a Golgi protein, is impaired. This observation is consistent with previous data from an Osteogenesis Imperfecta mouse model bearing the G610C substitution in the procollagen α2(I) chain whereby the resulting procollagen triple helix misfolding leads to an unusual form of cellular stress not linked to the conventional UPR [29].

Altered ER structures have been described as a consequence of defects in proteins involved in the ER/Golgi trafficking. Mutations affecting trafficking protein particle complex 2 (TRAPPC2 or Sedlin) and dymeclin, thought to have roles in protein transport between ER and Golgi, also cause nonlethal skeletal dysplasias, X-linked spondyloepiphyseal dysplasia tarda and Dyggve-Melchior-Clausen syndrome, respectively [[30], [31], [32]]. Therefore, ER swelling does not result exclusively from structural defects in the secreted matrix proteins [18], but also from defects in the components of the secretory pathway and in the cross-talk between ER and Golgi. Interestingly, ER stress has not been described in sedlin or dymeclin mutant cells [30,31].

Chondrocytes produce several highly modified PGs, the most abundant of which is aggrecan, a very large macromolecule that contains more than a hundred GAG chains whose synthesis occurs in the Golgi [19,33]. Therefore, it is not surprising that defects in GAG synthesis may result in defects in organelle autoregulation leading to altered organelle structures.

### Role of glycosaminoglycan defects in DBQD1 pathology

The finding of defective GAG synthesis in the pathogenesis of DBQD1 comports well with the current understanding of cartilage function. In normal cartilage, the chondrocytes synthesise and deposit large amounts of sulphated PGs in the extracellular matrix, where these macromolecules are crucial for the mechanical and biological functions of cartilage.

PGs constitute a major regulatory point of chondrocyte activity providing structural support to chondrocytes and creating a milieu, which affects diffusion of growth factors, signaling molecules and nutrients [33,34]. It is well known that structural defects in the ECM macromolecules affect the properties of tissues, including cartilage. Interestingly, in transgenic mice expressing a mutated form of thyroglobulin, a protein not expressed in the growth plate, it has been demonstrated that mutant protein retention and intracellular stress *per se* directly disturb chondrocyte performance without altering secretion and assembly of ECM proteins [35]. This observation suggests that cell performance is not only affected by structural defects in ECM proteins, but also by proteins (including enzymes and transporters) involved in post-translational modifications, folding and quality control. Furthermore, increased post-translational modifications and delayed type I collagen secretion have been demonstrated in recessive forms of Osteogenesis Imperfecta, where the enzymes involved in collagen folding and post-translational modifications are defective (*i.e.* cartilage associated protein, prolyl 3-hydroxylase, serpin H1 and FKBP65) [26,36]. We therefore postulate that the pathomolecular mechanism of DBQD1 comprises of an intracellular (GAG synthesis defects, ER/Golgi enlargement and impaired PG secretion) and extracellular (PG structural defects) component.

It is interesting to note that the clinical abnormalities in DBQD1 are restricted to cartilage and bone, even though *CANT1* appears to be widely expressed. Cartilage-producing cells are thought to have a vastly greater requirement of sugars for PG synthesis than any other tissue [37] and the physiological consequences of altered GAG synthesis in such tissue may be much more profound. However, we cannot exclude that other tissues may have alternative strategies for their modest Golgi glycosylation requirements, including different nucleotidases.

To conclude, the *Cant1*^*−/−*^ mouse is an appropriate animal model of DBQD1 and it recapitulates the typical clinical features observed in DBQD1 patients. Moreover, GAG synthesis is decreased and PG secretion is reduced in mutant cartilage, demonstrating that CANT1 plays a role in PG metabolism. The PG defects cause deregulated chondrocyte proliferation and maturation in the cartilage growth plate. Further studies of the cellular pathways involved in the defective ER Golgi cross-talk and organelle autoregulation will be crucial in order to elucidate the molecular basis of the disease and to pave the way for developing therapeutic strategies for this rare chondrodysplasia.

## Materials and methods

### Preparation of the gene targeting vector

The short and long arm of the gene targeting vector were generated by PCR amplification of the *Cant1* gene targeting vector CANT1 PG00123_Y_1_H12 from Helmholtz Zentrum Munchen, Germany (https://www.eummcr.org). The sequence of the gene targeting vector is available at https://www.i-dcc.org/imits/targ_rep/alleles/10068/targeting-vector-genbank-file. The main components of the vector are the 5′ and 3′ homology arms that mediate homologous recombination at the murine *Cant1* locus and a central targeting cassette that disrupts gene function and reports gene expression with a lacZ reporter. The targeting cassette is flanked by FRT recombination sites, which allows the removal of the targeting cassette with Flp recombinase. In addition, exon 3 and 4 of the murine *Cant1* gene are flanked by a pair of loxP recombination sites for conditional gene inactivation by deleting the two exons with Cre recombinase.

The 5′arm containing the 5′ Cant1 homologous region, exon 2 and an FRT recombination site was amplified by high fidelity PCR (PfuUltra II fusion HS DNA Polymerase, Agilent Technologies). The sense and antisense primers were designed according to the nucleotide sequence of the CANT1 PG00123_Y_1_H12 plasmid vector and contained the *HindIII* restriction site (5′-AGCAGG**AAGCTT**TGACTTGGTGAGGCTCCC-3′ and 5′-TCACAA**AAGCTT**CTTCTGTTAGTCCCAACCC-3′, respectively). The amplified 4.1 kb 5′ arm after *HindIII* (Promega) digestion was cloned in the *HindIII* multiple cloning site 1 of the NTKV1902 vector (pKO Scrambler NTKV, Stratagene), a gene targeting vector containing the herpes simplex *thymidine kinase* (*Tk*) gene, driven by the polyoma virus thymidine kinase (MC1) promoter, and *neomycin* gene, driven by the phosphoglycerate kinase (PGK) promoter, for negative and positive selection, respectively.

Likewise, the 3′arm was amplified by high fidelity PCR using sense and antisense primers containing the *SacII* restriction site (5′-CTGGAT**CCGCGG**GTACCGCGTCG-3′ and 5′-ACATGA**CCGCGG**CTGCTTCTGGGGTTGG-3′, respectively). Then the 7.5 kb 3′arm containing a FRT site and exon 3 and 4 flanked by loxP sites was cloned in pBluescript II KS (pBS3′arm). The c.905G > A transition causing the R302H substitution in *Cant1* was introduced in the pBS3’arm by site-directed mutagenesis with the QuickChange II XL Site-Directed Mutagenesis Kit (Agilent Technologies), according to the manufacturer instructions. Finally, the mutated 3’arm was cloned in the *SacII* restriction site of the multiple cloning site 2 of the gene targeting vector.

### Generation of Cant1 targeted embryonic stem cells and mice

Electroporation of the linearized targeting vector (Fig. 1A) into ES cells with C57Bl/6J × 129/SV background was performed by the Core Facility for Conditional Mutagenesis (Dibit, San Raffaele Hospital, Milan, Italy) and was followed by transfected ES cells exposure to G418 and ganciclovir. Selected colonies were isolated and screened by Southern blot analysis for the recombinant allele. Correct 5′ targeting was confirmed by DNA digestion with *ScaI* and membrane hybridization with a PCR probe spanning *Cant1* exon 2. Correct 3′ targeting was confirmed by DNA digestion with *KpnI* and membrane hybridization with a PCR probe spanning a *Cant1* exon 4 fragment. The 5′ end probe detected a 13.9 kb *ScaI* fragment specific for the targeting allele and a 11.6 kb fragment for the wild-type allele; the 3′ end probe detected a 12.8 kb *KpnI* fragment for the targeted allele and a 14.2 kb fragment for the wild-type allele (Fig. 1B). The presence of the mutation was confirmed by direct sequencing and two positive clones were injected in C57Bl/6J × 129/SV mouse blastocysts. The resulting chimeric male mice were mated with a Flp recombinase transgenic strain (B6.129S4-Gt(ROSA)26Sor^tm1(FLP1)Dym^, Jackson Laboratory) to delete the frt-flanked neomycin selection cassette. Offspring heterozygous for the mutation were used to generate the *Cant1* p.R302H knock-in mice (*Cant1*^R302H/R302H^).

To generate the *Cant1* knock-out strain (*Cant1*^−/−^), heterozygous knock-in animals were mated with a Cre mouse strain (B6.FVB-Tg(EIIa-cre)C5379Lmgd) to delete exon 3 and 4 that were flanked by the loxP recombination sites (Fig. 1C).

### Animal care

Animals were bred with free access to water and standard pelleted food. Care and use of mice complied with relevant animal welfare institutional guidelines and protocols were approved by the Animal Care and Use Committee of the University of Pavia and the Ministry of Health (Licence n. 95/2017-PR).

### Mouse genotyping.

Mice were genotyped by PCR using genomic DNA from mouse tail clips. PCR primers to genotype homozygous knock-in mice (*Cant1*^R302H/R302H^ mice) from heterozygous and wild type animals were CAN18 (5’-CCTGTGGAGGTTGGGATTCC- 3′) and CAN47 (5’-CAAATGAGGCCCAGGAAGTG- 3′), whilst CAN18, CAN47 and CANFRT3 (5’-AAATGATCACTGCCTTGTCCTG- 3′) primers were used for genotyping homozygous knock-out mice (*Cant1*^−/−^ mice) from heterozygous and wild type animals.

### Real time PCR

Total RNA was extracted from skin by QIAzol® Lysis Reagent (QIAGEN) according to manufacturer instructions. RNA (2 μg) was reverse transcribed using the High Capacity cDNA Reverse Transcription kit (Applied Biosystems) following manufacturer's recommendation. Relative quantitative real time PCR experiments were performed using the QuantiFast SYBR Green PCR Kit (QIAGEN) with QuantiTect Primer Assay (QIAGEN) for *Cant1* (QT01041789) and TATA box binding protein (QT00198443) as housekeeping gene for expression normalization. Each sample was run in triplicate in 96 well plates in three independent experiments with the MX3000P (Stratagene) apparatus. Three mice for each genotype were analysed and relative *Cant1* expression was determined with the ΔΔCt method.

### Skeletal staining with alcian blue and alizarin red

Skeletal characterization of mice was performed by double staining with alcian blue and alizarin red to stain cartilage and bone, respectively, as described previously [38]. Images of skeletal preparations were acquired by a Leica DFC425 C digital camera connected to a Leica M165 FC stereomicroscope. Skeletal morphometric analysis were performed using the LAS 4.5 software (Leica) by two different observers blinded to genotype.

### X-ray analysis

A Faxitron MX-20 cabinet X-ray system (Faxitron Bioptics) was used for X-ray images of mice. The exposure was set to 27 kV for 19 s with 2-fold magnification for adult mice. Kodak DirectView Elite CR System (Carestream Health) was used to capture X-ray images.

### Histological staining and morphometric analysis

For histological study, hind limbs were dissected immediately after sacrifice, fixed with 10% formalin in PBS (Sigma-Aldrich) and processed for light microscopy according to standard procedures after decalcification in 14% ethylenediaminetetraacetic acid (EDTA) pH 7.1 at room temperature for 2–4 weeks depending on the age of the mice.

For histomorphometric analysis, slides were stained with toluidine blue as described previously [21] and images of sections were acquired using a DM5500 B microscope (Leica) connected to a Leica DFC 480 camera. All morphometric measurement were performed by LAS V4.5 software (Leica). The resting, proliferative and hypertrophic zones were defined on the basis of cell morphology as reported in literature [39]. To determine the height of each zone at least five measurements per zone per section were performed. To determine the number of cells per column and the height of columns in different zones at least 20 columns per zone per sections were analysed. An average of five sections per animal was considered. All measurements were performed by two different observers blinded to genotype.

### Proliferation and apoptosis analysis

For immunohistochemical detection of proliferative cells in the growth plate, mice were intraperitoneally injected with 100 mg/kg 5′-bromo-2′-deoxyuridine (BrdU) (Sigma-Aldrich) as previously reported [39]. Mice were sacrificed 2 h post injection; hind limbs were harvested and processed according to standard procedure. 6 μm sections were analysed using the BrdU Staining Kit (Invitrogen) according to the manufacturer suggestions. Images of sections were acquired using a DM5500 B microscope (Leica) connected to a Leica DFC 480 camera. All measurements were performed using LAS V4.5 software (Leica).

For the immunohistochemical detection of apoptotic chondrocytes in the growth plate, the DeadEnd™ Fluorometric TUNEL System (Promega) was used on 6 μm sections according to manufacturer's instructions. Images of sections were captured by a fluorescence microscope Axioimager.Z2 (Zeiss) and all measurements were performed using Image J software.

Proliferating and apoptotic cell measurements were performed by two different observers blinded to genotype.

### Primary chondrocyte cultures

To establish primary chondrocyte cultures, thoracic cage of P4 mice was harvested and digested with 2 mg/ml collagenase type II (Invitrogen) in Dulbecco's modified Eagle's medium (DMEM) (Sigma-Aldrich) at 37 °C for 90 min. The cartilage was dissected from each rib under the microscope and digested with 2 mg/ml collagenase type II at 37 °C for 3 h. The released chondrocytes were plated and cultured in DMEM with 10% foetal calf serum (FCS) (EuroClone) at 37 °C in a humidified atmosphere containing 5% CO_2_.

### Metabolic labelling of chondrocyte cultures and PG synthesis analysis

Chondrocyte cultures were preincubated with or without 1 mM *p*-nitrophenyl β-d-xylopyranoside (Sigma-Aldrich) in minimal essential medium (MEM) (Sigma-Aldrich) containing 250 μM cold Na_2_SO_4_ without FCS at 37 °C in 5% CO_2_ for 2 h. Cells were then labelled with 50 μCi/ml Na_2_[^35^SO_4_] (38.8–59.2 TBq/mmol, PerkinElmer) in the same medium for 24 h as described previously [40]. At the end of the labelling period, an equal volume of 100 mM sodium acetate buffer, pH 5.8, containing 8 M urea, 4% Triton X-100, 20 mM EDTA, 20 mM N-ethylmalemide (NEM), 0.1 M 6-aminocaproic acid and 1 mM phenylmethylsulfonyl fluoride (PMSF) was added to the medium. The cell layer was lysed in 50 mM sodium acetate buffer, pH 5.8, containing 2 M urea and 2% Triton X-100, an aliquot was used to determine the protein content by the BCA Protein Assay (Pierce) and the rest was added to the medium. Samples were loaded on 1 ml DEAE Sephacel columns and, after column washing with 50 mM sodium acetate, pH 6.0, containing 8 M urea, 0.15 M NaCl, 0.5% Triton X-100, 10 mM EDTA, 10 mM NEM, 0.1 M 6-aminocaproic acid and 0.5 mM PMSF, PGs were eluted with 1 M NaCl in the same buffer. The PGs were then precipitated with 9 volumes of 96% ethanol at 4 °C overnight and centrifuged at 17,300 ×*g* at 4 °C for 50 min. The pellet was washed with 70% ethanol and then solubilized in water. PGs were quantified by measuring the ^35^S-activity using a liquid scintillation counter and normalised to the protein content.

### Size exclusion chromatography of GAG chain

Labelled PGs synthesised by chondrocytes and purified as described above were β-eliminated by alkaline digestion with 0.125 M NaOH followed by reduction with 1 M NaBH_4_ at room temperature overnight to release GAG chains. After neutralization with acetic acid, samples were lyophilized and dissolved in 50 mM sodium acetate, pH 6.0, containing 4 M guanidinium chloride (GuHCl) and 0.5% Triton X-100. Samples were loaded on a Superose 6 10/300GL column (GE Healthcare) and eluted in the same buffer at 0.2 ml/min. Fraction of 0.4 ml were collected and ^35^S-activity was measured by scintillation counting.

### Analysis of PG secretion

In a pulse chase experiment, primary chondrocytes were labelled with MEM without FCS containing 100 μCi/ml Na_2_[^35^SO_4_] at 37 °C in 5% CO_2_ for 2 h. At the end of the labelling period, the medium was removed, cells were washed with MEM without FCS containing 2.5 mM cold sulfate and incubated in the same medium for different chase times (0, 0.5, 2.5 and 5 h) as described previously [41]. At each time point, the cell layer and medium were collected separately. To each medium the same volume of 0.2 M sodium acetate, pH 5.8, containing 8 M GuHCl, 2% Triton X-100 and protease inhibitors (4 mM EDTA, 10 mM benzamidine, 1.9 mM NEM) was added, whilst the cell layer was scraped in 0.1 M sodium acetate, pH 5.8, containing 4 M GuHCl, 2% Triton X-100 and protease inhibitors. Each medium and cell layer were desalted to remove free ^35^S-sulfate with PD Miditrap G-25 columns (GE Healthcare) equilibrated and eluted with 50 mM sodium acetate, pH 6.0, containing 4 M GuHCl and 0.5% Triton X-100. The V_0_ from each gel filtration column was collected and ^35^S-activity was measured by scintillation counting. For each time point, the percentage of ^35^S-activity in the medium to the total counts (medium and cell layer) was calculated.

### Glycosaminoglycan content assay

GAG content was measured in the femoral head cartilage of P4 mice using the dimethylmethylene blue (DMMB) assay [42]. Briefly, femoral head cartilages were dissected and digested with 300 μl of 1.67 mg/ml proteinase K (Sigma-Aldrich) in 0.1 M ammonium acetate, pH 7.35, containing 5 mM EDTA at 55 °C overnight. Then samples were incubated at 100 °C for 10 min to denature proteinase K. The DMMB assay was performed on 60 μl of digested samples adding 1 ml of DMMB solution (10.7 mg/l DMMB (Sigma-Aldrich) in 55 mM formic acid, pH 3.3). Sample absorbance was read at 520 nm immediately after DMMB addition. To determine the GAG concentration, a standard curve containing up to 6 μg chondroitin sulfate in the sample buffer was used. GAG content was normalised to DNA amount analysed on digested samples by the Quant-iT™ PicoGreen® dsDNA Assay Kit (Invitrogen), according to the manufacturer's instructions.

### Proteoglycan sulfation analysis

For PG sulfation analysis, GAGs were extracted from cartilage and primary chondrocyte cultures. GAGs from femoral head cartilage of P4 mice were obtained by proteinase K digestion of the tissue as described above. Chondrocyte cultures were incubated with basal medium without FCS at 37 °C in 5% CO_2_ for 24 h. Then, the medium was made 0.1 M sodium acetate, pH 5.6, 5 mM EDTA and 5 mM cysteine and 20 U of papain (Sigma) were added, while the cell layer was scraped in papain digestion buffer (0.1 M sodium acetate, pH 5.6, 5 mM EDTA and 5 mM cysteine) and digested with 20 U of papain. Digestion was performed at 65 °C overnight.

Papain and proteinase K in all digested samples (from cell cultures or from femoral head cartilage) were inactivated at 100 °C for 10 min and released GAGs were recovered and analysed by HPLC after 2-aminoacridone derivatization as previously described [43].

### Transmission electron microscopy analysis

Femoral head cartilages were processed for transmission electron microscopy as reported previously [44]. Briefly, cartilage was dissected and fixed with 2% (v/v) glutaraldehyde and 0.7% (w/v) ruthenium hexamine trichloride (RHT) in 0.1 M cacodylate buffer, pH 7.4, at room temperature for 3 h. The samples were then washed with 0.7% RHT in 0.1 M cacodylate buffer, pH 7.4. Samples were post-fixed in 0.7% RHT and 2% (w/v) osmium tetroxide (OsO_4_) in 0.1 M cacodylate buffer at room temperature for 2 h, rinsed in distilled water, dehydrated in ethanol and infiltrated with LR White acrylic resin. Thin sections (60–70 nm tick) were cut on a Reichert OM-U3 ultramicrotome, stained with saturated aqueous uranyl acetate followed by lead citrate and observed with a Zeiss EM900 electron microscope at 80 kV.

### Microarray analysis

Total RNA was extracted from rib cartilage for microarray analysis. Thoracic cage of P4 mice was dissected, digested with 2 mg/ml collagenase type II in DMEM at 37 °C for 90 min and costal cartilages were harvested from each rib under the dissection microscope. The RNA then was purified from costal cartilage using RNeasy Plus Mini kit (QIAGEN) according to manufacturer's protocol. RNA quality was analysed by Agilent 2100 Bioanalyzer (Agilent Technologies) using the RNA Nano chip kit (Agilent Technologies), while RNA concentration was determined by the NanoDrop ND-1000 spectrophotometer.

The GeneChip® Mouse Genome 430A 2.0 microarray (Affymetrix) was used and processed with the GeneChip® 3’ IVT PLUS Reagent Kit and the GeneChip® Hybridisation Wash and Stain Kit. The microarray analysis was performed by the Center for Genome Research of the University of Modena and Reggio Emilia, Italy. The fold change threshold between *Cant1*^−/−^ and *Cant1*^+/+^ mice was set ±2.

### RT-PCR analysis

Total RNA was extracted from chondrocytes by QIAzol® Lysis Reagent (QIAGEN) according to manufacturer instructions. Then cDNA was obtained from purified RNA by SuperScript™ IV First-Strand Synthesis System (Invitrogen) in accordance with manufacturer's protocol. The presence of the Xbp1 spliced form was analysed by RT-PCR as described in literature [16] using the following primers: XBP1 forward (5′-GAACCAGGAGTTAAGAACACG- 3′) and XBP1 reverse (5′-AGGCAACAGTGTCAGAGTCC- 3′).

Briefly, 5 μl cDNA was subjected to PCR as follows: 3 min at 95 °C, followed by 35 cycles of 95 °C for 40 s, 60 °C for 45 s and 72 °C for 40 s and then 10 min of extension at 72 °C. PCR products were separated by electrophoresis on 8% acrylamide gel in TBE buffer. The Xbp1 unspliced form band was 205 bp long, while the Xbp1 spliced form band was 179 bp long.

### Western blot analysis

For western blot analysis, 2 × 10^6^ chondrocytes were plated in 60 mm diameter culture dishes with DMEM and 10% FCS and incubated at 37 °C in 5% CO_2_. After 3 days, the cells were scraped in PBS and centrifuged at 1,200 ×*g* for 15 min. The cell pellets were lysed and analysed by western blot as previously described [45]. SDS-PAGE was carried out on 10% polyacrylamide gels. Primary antibodies to BiP (rabbit monoclonal antibody, Cell Signaling), to ATF-4 (rabbit monoclonal antibody, Cell Signaling), to actin (goat polyclonal antibody, Santa Cruz Biotechnology) and the appropriate HRP secondary antibody (goat anti-rabbit polyclonal antibody, Cell Signaling and donkey anti-goat polyclonal antibody, Santa Cruz Biotechnology) were used. The ImageQuant TL software was used for densitometry analysis and the intensity of the wild type band was set to 1 and the expression of mutant samples was expressed as fold change.

### Statistical analysis

Statistical analysis was performed using Microsoft Excel software. All values are expressed as mean ± standard deviation (SD). Statistical difference between different groups was evaluated using Student's *t*-test and a *p*-value < 0.05 was considered statistically significant.

## Appendix A Supplementary data

Supplementary material Image 1

## References

[1] Bulow H.E., Hobert O. (2006). The molecular diversity of glycosaminoglycans shapes animal development. Annu. Rev. Cell Dev. Biol..

[2] Vynios D.H. (2014). Metabolism of cartilage proteoglycans in health and disease. Biomed. Res. Int..

[3] Iozzo R.V., Schaefer L. (2015). Proteoglycan form and function: a comprehensive nomenclature of proteoglycans. Matrix Biol..

[4] Mizumoto S., Sugahara K., Nikos K. (2012). Bone and skin disorders caused by a disturbance in the biosynthesis of chondroitin sulfate and dermatan sulfate. Extracellular Matrix: Pathobiology and Signaling, Walter de Gruyter, Berlin, Germany.

[5] Mizumoto S., Yamada S., Sugahara K. (2014). Human genetic disorders and knockout mice deficient in glycosaminoglycan. Biomed. Res. Int..

[6] Bonafe L., Cormier-Daire V., Hall C., Lachman R., Mortier G., Mundlos S., Nishimura G., Sangiorgi L., Savarirayan R., Sillence D., Spranger J., Superti-Furga A., Warman M., Unger S. (2015). Nosology and classification of genetic skeletal disorders: 2015 revision. Am. J. Med. Genet. A.

[7] Faivre L., Cormier-Daire V., Eliott A.M., Field F., Munnich A., Maroteaux P., Le Merrer M., Lachman R. (2004). Desbuquois dysplasia, a reevaluation with abnormal and “normal” hands: radiographic manifestations. Am. J. Med. Genet. A.

[8] Huber C., Oules B., Bertoli M., Chami M., Fradin M., Alanay Y., Al-Gazali L.I., Ausems M.G., Bitoun P., Cavalcanti D.P., Krebs A., Le Merrer M., Mortier G., Shafeghati Y., Superti-Furga A., Robertson S.P., Le Goff C., Muda A.O., Paterlini-Brechot P., Munnich A., Cormier-Daire V. (2009). Identification of CANT1 mutations in Desbuquois dysplasia. Am. J. Hum. Genet..

[9] Bui C., Huber C., Tuysuz B., Alanay Y., Bole-Feysot C., Leroy J.G., Mortier G., Nitschke P., Munnich A., Cormier-Daire V. (2014). XYLT1 mutations in Desbuquois dysplasia type 2. Am. J. Hum. Genet..

[10] Failer B.U., Braun N., Zimmermann H. (2002). Cloning, expression, and functional characterization of a Ca(2+)-dependent endoplasmic reticulum nucleoside diphosphatase. J. Biol. Chem..

[11] Nizon M., Huber C., De Leonardis F., Merrina R., Forlino A., Fradin M., Tuysuz B., Abu-Libdeh B.Y., Alanay Y., Albrecht B., Al-Gazali L., Basaran S.Y., Clayton-Smith J., Desir J., Gill H., Greally M.T., Koparir E., van Maarle M.C., MacKay S., Mortier G., Morton J., Sillence D., Vilain C., Young I., Zerres K., Le Merrer M., Munnich A., Le Goff C., Rossi A., Cormier-Daire V. (2012). Further delineation of CANT1 phenotypic spectrum and demonstration of its role in proteoglycan synthesis. Hum. Mutat..

[12] Smith T.M., Kirley T.L. (2006). The calcium activated nucleotidases: a diverse family of soluble and membrane associated nucleotide hydrolyzing enzymes. Purinergic Signal.

[13] Smith T.M., Hicks-Berger C.A., Kim S., Kirley T.L. (2002). Cloning, expression, and characterization of a soluble calcium-activated nucleotidase, a human enzyme belonging to a new family of extracellular nucleotidases. Arch. Biochem. Biophys..

[14] Cali T., Fedrizzi L., Ottolini D., Gomez-Villafuertes R., Mellstrom B., Naranjo J.R., Carafoli E., Brini M. (2012). Ca2+−activated nucleotidase 1, a novel target gene for the transcriptional repressor DREAM (downstream regulatory element antagonist modulator), is involved in protein folding and degradation. J. Biol. Chem..

[15] Harden T.K., Sesma J.I., Fricks I.P., Lazarowski E.R. (2010). Signalling and pharmacological properties of the P2Y receptor. Acta Physiol (Oxford).

[16] Forouhan M., Mori K., Boot-Handford R.P. (2018). Paradoxical roles of ATF6α and ATF6β in modulating disease severity caused by mutations in collagen X. Matrix Biol..

[17] Hunziker E.B., Schenk R.K., Cruz-Orive L.M. (1987). Quantitation of chondrocyte performance in growth-plate cartilage during longitudinal bone growth. J. Bone Joint Surg. Am..

[18] Briggs M.D., Bell P.A., Pirog K.A. (2015). The utility of mouse models to provide information regarding the pathomolecular mechanisms in human genetic skeletal diseases: the emerging role of endoplasmic reticulum stress (review). Int. J. Mol. Med..

[19] Krishnan Y., Grodzinsky A.J. (2018). Cartilage diseases. Matrix Biol..

[20] Patterson S.E., Dealy C.N. (2014). Mechanisms and models of endoplasmic reticulum stress in chondrodysplasia. Dev. Dyn..

[21] Gualeni B., Facchini M., De Leonardis F., Tenni R., Cetta G., Viola M., Passi A., Superti-Furga A., Forlino A., Rossi A. (2010). Defective proteoglycan sulfation of the growth plate zones causes reduced chondrocyte proliferation via an altered Indian hedgehog signalling. Matrix Biol..

[22] Hiraoka S., Furuichi T., Nishimura G., Shibata S., Yanagishita M., Rimoin D.L., Superti-Furga A., Nikkels P.G., Ogawa M., Katsuyama K., Toyoda H., Kinoshita-Toyoda A., Ishida N., Isono K., Sanai Y., Cohn D.H., Koseki H., Ikegawa S. (2007). Nucleotide-sugar transporter SLC35D1 is critical to chondroitin sulfate synthesis in cartilage and skeletal development in mouse and human. Nat. Med..

[23] Wilson D.G., Phamluong K., Lin W.Y., Barck K., Carano R.A., Diehl L., Peterson A.S., Martin F., Solloway M.J. (2012). Chondroitin sulfate synthase 1 (Chsy1) is required for bone development and digit patterning. Dev. Biol..

[24] Kluppel M., Wight T.N., Chan C., Hinek A., Wrana J.L. (2005). Maintenance of chondroitin sulfation balance by chondroitin-4-sulfotransferase 1 is required for chondrocyte development and growth factor signaling during cartilage morphogenesis. Development.

[25] Schwartz N.B., Domowicz M. (2002). Chondrodysplasias due to proteoglycan defects. Glycobiology.

[26] Marini J.C., Forlino A., Bachinger H.P., Bishop N.J., Byers P.H., Paepe A., Fassier F., Fratzl-Zelman N., Kozloff K.M., Krakow D., Montpetit K., Semler O. (2017). Osteogenesis imperfecta. Nat Rev Dis Primers.

[27] Morello R. (2018). Osteogenesis imperfecta and therapeutics. Matrix Biol..

[28] Lewy T.G., Grabowski J.M., Bloom M.E. (2017). BiP: master regulator of the unfolded protein response and crucial factor in Flavivirus biology. Yale J Biol Med.

[29] Mirigian L.S., Makareeva E., Mertz E.L., Omari S., Roberts-Pilgrim A.M., Oestreich A.K., Phillips C.L., Leikin S. (2016). Osteoblast malfunction caused by cell stress response to procollagen Misfolding in α2(I)-G610C mouse model of osteogenesis imperfecta. J. Bone Miner. Res..

[30] Osipovich A.B., Jennings J.L., Lin Q., Link A.J., Ruley H.E. (2008). Dyggve-Melchior-Clausen syndrome: chondrodysplasia resulting from defects in intracellular vesicle traffic. Proc. Natl. Acad. Sci. U. S. A..

[31] Venditti R., Scanu T., Santoro M., Di Tullio G., Spaar A., Gaibisso R., Beznoussenko G.V., Mironov A.A., Mironov A., Zelante L., Piemontese M.R., Notarangelo A., Malhotra V., Vertel B.M., Wilson C., De Matteis M.A. (2012). Sedlin controls the ER export of procollagen by regulating the Sar1 cycle. Science.

[32] Tiller G.E., Hannig V.L., Dozier D., Carrel L., Trevarthen K.C., Wilcox W.R., Mundlos S., Haines J.L., Gedeon A.K., Gecz J. (2001). A recurrent RNA-splicing mutation in the SEDL gene causes X-linked spondyloepiphyseal dysplasia tarda. Am. J. Hum. Genet..

[33] Kiani C., Chen L., Wu Y.J., Yee A.J., Yang B.B. (2002). Structure and function of aggrecan. Cell Res..

[34] Domowicz M.S., Cortes M., Henry J.G., Schwartz N.B. (2009). Aggrecan modulation of growth plate morphogenesis. Dev. Biol..

[35] Gualeni B., Rajpar M.H., Kellogg A., Bell P.A., Arvan P., Boot-Handford R.P., Briggs M.D. (2013). A novel transgenic mouse model of growth plate dysplasia reveals that decreased chondrocyte proliferation due to chronic ER stress is a key factor in reduced bone growth. Dis. Model. Mech..

[36] Morello R., Bertin T.K., Chen Y., Hicks J., Tonachini L., Monticone M., Castagnola P., Rauch F., Glorieux F.H., Vranka J., Bachinger H.P., Pace J.M., Schwarze U., Byers P.H., Weis M., Fernandes R.J., Eyre D.R., Yao Z., Boyce B.F., Lee B. (2006). CRTAP is required for prolyl 3-hydroxylation and mutations cause recessive osteogenesis imperfecta. Cell.

[37] Archer C.W., Francis-West P. (2003). The chondrocyte. Int. J. Biochem. Cell Biol..

[38] Forlino A., Piazza R., Tiveron C., Della Torre S., Tatangelo L., Bonafe L., Gualeni B., Romano A., Pecora F., Superti-Furga A., Cetta G., Rossi A. (2005). A diastrophic dysplasia sulfate transporter (SLC26A2) mutant mouse: morphological and biochemical characterization of the resulting chondrodysplasia phenotype. Hum. Mol. Genet..

[39] Vanky P., Brockstedt U., Hjerpe A., Wikstrom B. (1998). Kinetic studies on epiphyseal growth cartilage in the normal mouse. Bone.

[40] Rossi A., Kaitila I., Wilcox W.R., Rimoin D.L., Steinmann B., Cetta G., Superti-Furga A. (1998). Proteoglycan sulfation in cartilage and cell cultures from patients with sulfate transporter chondrodysplasias: relationship to clinical severity and indications on the role of intracellular sulfate production. Matrix Biol..

[41] Pacifici M. (1990). Independent secretion of proteoglycans and collagens in chick chondrocyte cultures during acute ascorbic acid treatment. Biochem. J..

[42] de Lima C.R., Baccarin R.Y., Michelacci Y.M. (2007). Reliability of 1,9-dimethylmethylene blue tests in comparison to agarose gel electrophoresis for quantification of urinary glycosaminoglycans. Clin. Chim. Acta.

[43] Monti L., Paganini C., Lecci S., De Leonardis F., Hay E., Cohen-Solal M., Villani S., Superti-Furga A., Tenni R., Forlino A., Rossi A. (2015). *N*-acetylcysteine treatment ameliorates the skeletal phenotype of a mouse model of diastrophic dysplasia. Hum. Mol. Genet..

[44] Hunziker E.B., Herrmann W., Schenk R.K. (1983). Ruthenium hexammine trichloride (RHT)-mediated interaction between plasmalemmal components and pericellular matrix proteoglycans is responsible for the preservation of chondrocytic plasma membranes in situ during cartilage fixation. J. Histochem. Cytochem..

[45] Besio R., Iula G., Garibaldi N., Cipolla L., Sabbioneda S., Biggiogera M., Marini J.C., Rossi A., Forlino A. (2018). 4-PBA ameliorates cellular homeostasis in fibroblasts from osteogenesis imperfecta patients by enhancing autophagy and stimulating protein secretion. Biochim. Biophys. Acta, Mol. Basis Dis..

