# Supplementary material for: Calcium activated nucleotidase 1 (CANT1) is critical for glycosaminoglycan biosynthesis in cartilage and endochondral ossification
Source: Matrix Biol. 2019 Aug;81:70–90. doi: 10.1016/j.matbio.2018.11.002 (PMC6598859; doi:10.1016/j.matbio.2018.11.002)
Supplement: Supplementary file 1 — Supplementary material [file mmc1.docx]

**SUPPLEMENTARY MATERIALS**

**Calcium activated nucleotidase 1 (CANT1) is critical for glycosaminoglycan biosynthesis in cartilage and endochondral ossification**

Chiara Paganini, Luca Monti, Rossella Costantini, Roberta Besio, Silvia Lecci, Marco Biggiogera, Kun Tian, Jean-Marc Schwartz, Céline Huber, Valérie Cormier-Daire, Beth G. Gibson, Katarzyna A. Pirog, Antonella Forlino and Antonio Rossi

**Supplementary Figure 1**
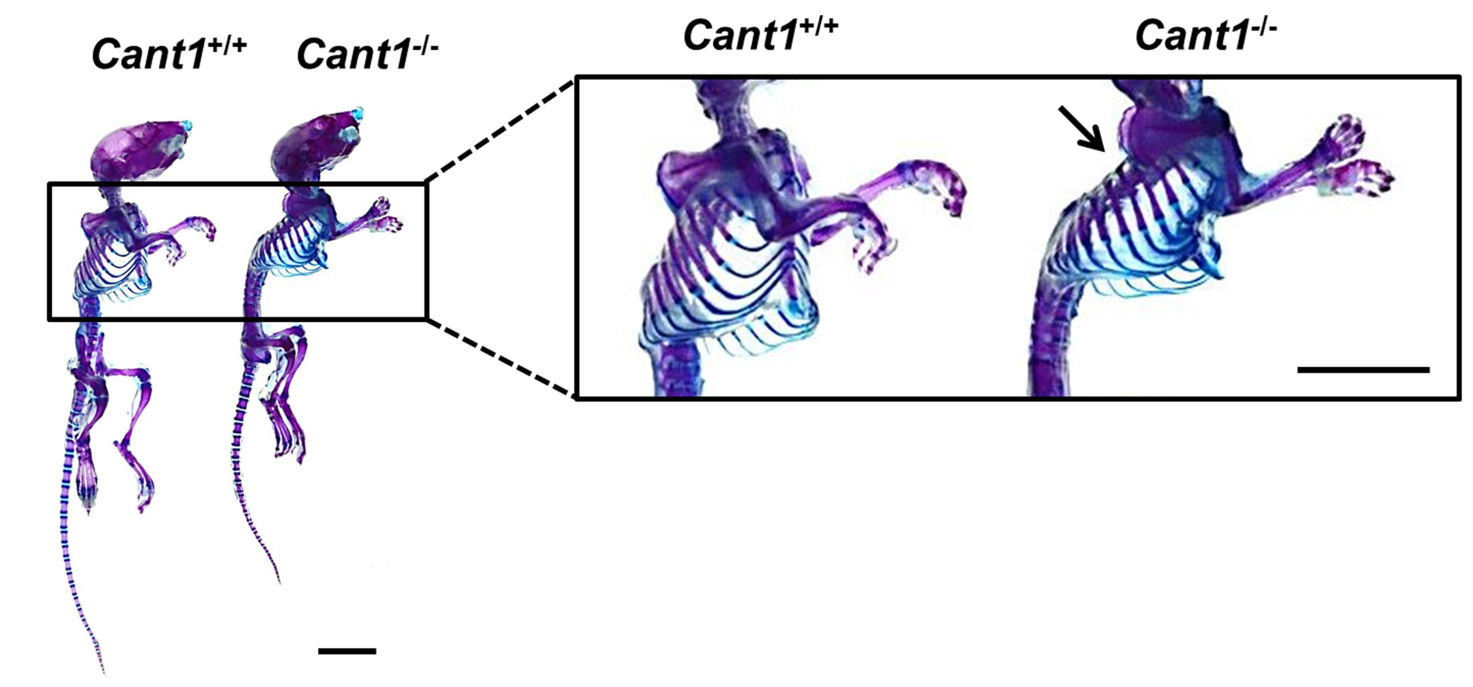


**Supplementary Figure 1.** Moderate thoracic kyphosis is present in *Cant1*^-/-^ mice. Skeletal preparations stained with alcian blue and alizarin red of P21 *Cant1*^-/-^ and *Cant1*^+/+^ mice showed the presence of moderate thoracic kyphosis (arrow) in mutant mouse. Scale bar = 1 cm.

## Supplementary Table 1

## Microarray expression analysis of chondroitin and heparan sulfotransferases.

| gene and/or protein name | gene symbol | Fold change (*Cant1*^-/-^ vs *Cant1*^+/+^) |
| --- | --- | --- |
| Carbohydrate (chondroitin 6/keratan) sulfotransferase 3 | *Chst3* | 1.04 |
| Carbohydrate (N-acetylglucosamino) sulfotransferase 7 | *Chst7* | 1.80 |
| Carbohydrate (chondroitin 4) sulfotransferase 11 | *Chst11* | -1.50 |
| Carbohydrate sulfotransferase 12 | *Chst12* | -1.12 |
| Carbohydrate (N-acetylgalactosamine 4-O) sulfotransferase 14 | *Chst14* | 1.13 |
| Carbohydrate (N-acetylgalactosamine 4-sulfate 6-O) sulfotransferase 15 | *Chst15* | -1.12 |
| Heparan sulfate 2-O-sulfotransferase 1 | *Hs2st1* | 1.13 |
| Heparan sulfate (glucosamine) 3-O-sulfotransferase 1 | *Hs3st1* | 1.67 |
| Heparan sulfate (glucosamine) 3-O-sulfotransferase 2 | *Hs3st2* | 1.07 |
| Heparan sulfate (glucosamine) 3-O-sulfotransferase 3A1 | *Hs3st3a1* | -1.19 |
| Heparan sulfate (glucosamine) 3-O-sulfotransferase 3B1 | *Hs3st3b1* | -1.17 |
| Heparan sulfate (glucosamine) 3-O-sulfotransferase 6 | *Hs3st6* | 1.09 |
| N-deacetylase/N-sulfotransferase (heparan glucosaminyl) 1 | *Ndst1* | 1.33 |
| N-deacetylase/N-sulfotransferase (heparan glucosaminyl) 2 | *Ndst2* | 1.01 |
| N-deacetylase/N-sulfotransferase (heparan glucosaminyl) 3 | *Ndst3* | -1.04 |
| N-deacetylase/N-sulfotransferase (heparan glucosaminyl) 4 | *Ndst4* | 1.05 |

RNA from cartilage of three animals for each genotype was analysed. The fold change threshold was set to ± 2; the expression level of none of the gene satisfied the fixed threshold.

**Supplementary Table 2**

## Microarray expression analysis of transcription factors and chaperons involved in ER stress and UPR.

| gene and/or protein name | gene symbol | Fold change (*Cant1*^-/-^ vs *Cant1*^+/+^) |
| --- | --- | --- |
| Heat shock 70 kDa protein 5 (glucose-regulated protein BiP) | *Bip/Hspa5* | -1.17 |
| Activating transcription factor 4 | *Atf4* | -1.10 |
| Activating transcription factor 6 | *Atf6* | -1.01 |
| Serine/threonine-protein kinase/endoribonuclease IRE1 | *Ern1/Ire1* | 1.03 |
| X-box-binding protein 1 | *Xbp1/Treb5* | -1.10 |
| PRKR-like endoplasmic reticulum kinase | *Eif2ak3/Perk* | -1.27 |
| Heat shock protein 90 kDa beta (Grp94), member1 | Grp94/Hsp90b1 | -1.09 |
| DNA damage-inducible transcript 3 protein | *Ddit3/Chop* | -1.70 |
| Protein disulfide isomerase-associated 6 | *Pdia6/Txndc7* | -1.11 |
| Protein disulfide isomerase-associated 4 (Erp 72) | *Erp72/Pdia4* | -1.13 |
| DnaJ (Hsp40) homologue, subfamily A, member 4 | *Dnaja4* | -1.32 |
| DnaJ (Hsp40) homologue, subfamily C, member 3 | *Dnajc3/P58ipk* | -1.04 |
| DnaJ (Hsp40) homologue, subfamily B, member 1 | *Dnajb1/Hsp40* | -1.07 |
| Protein disulfide isomerase-associated 3 (Erp 57) | *Erp57/Pdia3* | 1.00 |
| Calnexin | *Canx* | 1.02 |
| Calreticulin | Calr | 1.07 |

RNA from cartilage of three animals for each genotype was analysed. The fold change threshold was set to ± 2; the expression level of none of the gene satisfied the fixed threshold.
